# An empirical method that separates irreversible stem radial growth from bark water content changes in trees: theory and case studies

**DOI:** 10.1111/pce.12863

**Published:** 2017-01-06

**Authors:** Maurizio Mencuccini, Yann Salmon, Patrick Mitchell, Teemu Hölttä, Brendan Choat, Patrick Meir, Anthony O'Grady, David Tissue, Roman Zweifel, Sanna Sevanto, Sebastian Pfautsch

**Affiliations:** ^1^ School of GeoSciences University of Edinburgh Edinburgh EH9 3JN UK; ^2^ CREAF Cerdanyola del Vallès Barcelona 08193 Spain; ^3^ ICREA Pg. Lluís Companys 23 Barcelona 08010 Spain; ^4^ Department of Physics University of Helsinki Helsinki 00014 Finland; ^5^ CSIRO Land and Water Hobart Tas. Australia; ^6^ Department of Forest Sciences University of Helsinki Helsinki 00014 Finland; ^7^ Hawkesbury Institute for the Environment Western Sydney University Richmond NSW 2753 Australia; ^8^ Research School of Biology Australian National University Canberra ACT 2601 Australia; ^9^ Swiss Federal Institute for Forest Snow and Landscape Research, (WSL) Birmensdorf 8903 Switzerland; ^10^ Earth and Environmental Sciences Division Los Alamos National Laboratory Los Alamos NM 87545 USA

**Keywords:** hydraulic capacitance, bark water use, plant water potential, stem dendrometry

## Abstract

Substantial uncertainty surrounds our knowledge of tree stem growth, with some of the most basic questions, such as when stem radial growth occurs through the daily cycle, still unanswered.

We employed high‐resolution point dendrometers, sap flow sensors, and developed theory and statistical approaches, to devise a novel method separating irreversible radial growth from elastic tension‐driven and elastic osmotically driven changes in bark water content. We tested this method using data from five case study species. Experimental manipulations, namely a field irrigation experiment on Scots pine and a stem girdling experiment on red forest gum trees, were used to validate the theory.

Time courses of stem radial growth following irrigation and stem girdling were consistent with *a‐priori* predictions. Patterns of stem radial growth varied across case studies, with growth occurring during the day and/or night, consistent with the available literature. Importantly, our approach provides a valuable alternative to existing methods, as it can be approximated by a simple empirical interpolation routine that derives irreversible radial growth using standard regression techniques. Our novel method provides an improved understanding of the relative source–sink carbon dynamics of tree stems at a sub‐daily time scale.

## Abstract 2

Our knowledge of the daily patterns of tree stem growth is limited. Sap flow sensors and stem dendrometers were used to isolate irreversible stem radial growth. New theory was developed that fully permits to separate elastic from irreversible diameter changes. Under drought conditions, growth was limited to night‐time. Under well‐watered conditions, growth tended to occur throughout the 24 h cycle, with differences from case study to case study.

## Introduction

Despite their importance for the global carbon cycle, we have limited knowledge of the temporal patterns of radial growth of tree stems, particularly at short time scales, such as hourly or daily (cf., De Swaef *et al.* 2015). Together with growth in height, growth in stem girth is responsible for the net accumulation of above‐ground carbon in forest ecosystems. This lack of mechanistic understanding limits our ability to represent tree growth in ecosystem and global scale models, as evidenced in a recent comparison of tree ring data against Coupled Model Intercomparison Project, Phase 5 (CMIP‐5) class models (Anderegg *et al.* 2015). Measurements of stem radial growth of trees using high‐temporal resolution sensors (point dendrometers) have been carried out for several decades (Klepper *et al.* 1971), giving insight into stem diameter changes at time scales from minutes to multiple years. However, it has not been possible to fully isolate radial growth from other co‐occurring periodic signals, because daily growth is generally obscured by changes caused by elastic bark and xylem contraction/swelling during the day/night periods (cf., Irvine & Grace 1997; Chan *et al.* 2015; Zweifel 2016; Zweifel *et al.* 2016).

The biophysical processes occurring during radial growth are only partially known at a molecular level. New xylem and phloem cells extend from the secondary cambium through a process of controlled creep (sometimes referred to as plastic, as opposed to viscoelastic, extension), in which cellulose microfibrils, hemicelluloses and pectines slide along the wall, increasing its surface area as a consequence of turgor pressure (Cosgrove 2005). When expansion ceases, a lignin‐rich secondary wall is laid down inside the primary wall, making it stronger. Much progress has been made at the molecular and cellular scale to understand the biology of cellular growth (e.g. Lastdrager *et al.* 2014). Yet, these processes are very poorly understood and represented at the whole‐plant scale, especially under environmentally stressful conditions. For the case studies of monocot and dicot leaf growth, cell division and tissue expansion are considered two relatively independent processes, with different responses to water deficit (e.g. Tardieu *et al.* 2012). Leaf tissue expansion is likely to be controlled by co‐ordinated changes in cell wall stiffening (via the expression of several expansin genes and proteins such as DELLA) and plant hydraulic conductance. This may or may not be coupled to changes in cell turgor, with significant evidence pointing instead to the importance of growth‐driven water potential gradients at constant turgor (reviewed by Tang & Boyer 2002). Overall, photosynthesis, C metabolism, cell wall mechanical properties, plant hydraulics and the cell cycle are the major processes involved in controlling growth under water deficit, with their action mediated by various hormones (reviewed by Tardieu *et al.* 2012). To these controls, one possibly needs to add potential phloem transport limitations in tall trees (e.g. Thompson 2006). It is generally assumed that stem radial growth occurs at night, when plant water status and cellular turgor are close to their maximum values (e.g. Steppe *et al.* 2015). It is also often postulated that radial growth depends on substrate availability determined by the rate of long‐distance phloem supply and locally available carbohydrates (e.g. Daudet *et al.* 2005; Hölttä *et al.* 2010). If that was the case, one would predict changes in bark diameter growth rates following predictable daily cycles, as a result of periodic changes in both plant water status and photosynthesis. No direct measurements that we are aware of are available to test this assumption for the case of tree radial growth.

To deal with the complexity of the processes involved in growth at various spatio‐temporal scales, one strategy has been to develop numerically complex and parameter‐rich models (e.g. Steppe *et al.* 2006; Hölttä *et al.* 2010; de Schepper & Steppe 2010; De Swaef *et al.* 2013a and review by De Swaef *et al.* 2015), which simulate all component processes including growth. One disadvantage of this approach is that some of the growth‐related parameters are only known at the scale of individual cells. Because they cannot be estimated from available increment data, they need to be set *a priori*. As a consequence, it is difficult both to determine general diel patterns of tree growth and to identify critically limiting variables. An alternative strategy has been to develop empirical fitting schemes calculating various indices of daily and seasonal growth rates to relate them to environmental factors (e.g Deslauriers *et al.* 2003; Zweifel *et al.* 2016; van der Maaten *et al.* 2016). These approaches are also useful and provide valuable ecological information. However, they do not extract the necessary mechanistic components specifying growth dynamics at the sub‐daily scale.

Here, our objective is to develop theory and test a simple empirical interpolation routine that derives irreversible radial growth from diurnal stem diameter variation measurements. To accomplish this objective, we: (1) Use a model of radial water transfer (Mencuccini *et al.* 2013; Chan *et al.* 2015) to compute the changes in tree diameter caused by the use of bark‐stored water from the raw diameter readings. (2) Combine this approach with a model of bark osmotic content to separate osmotic‐related elastic changes from irreversible growth. (3) Employ this dual approach to separate observed daily patterns of bark contraction/swelling into three processes, i.e. (a) bark water content changes linked to transpiration‐driven xylem negative pressure (for simplicity, tension, from now on), (b) bark water content changes linked to osmotic‐related elastic changes and (c) actual growth. Growth is defined here as the irreversible addition of new cells or the irreversible expansion of existing cells. Finally, (4) we test our model using two field and one greenhouse experiment on five tree species. The first experiment tested the model by comparing the diel pattern of diameter growth of irrigated and control Scots pine (*Pinus sylvestris* L.) trees in a field experiment in Switzerland. We hypothesized that irrigation changed the diel pattern of growth. In particular, as a consequence of reductions in cellular turgor and carbon availability, growth would be limited to night‐time in the control plot; however, improvement in water supply and carbon status would extend growth into the daytime in the irrigated plot. The second experiment tested the model by comparing the diel pattern of diameter growth of girdled and control forest red gum (*Eucalyptus tereticornis* Sm.) trees in a field experiment with well‐watered trees in Australia. Here, we hypothesized that relative to the growth pattern prior to girdling, increased substrate availability above the point of girdling would result in growth continuing throughout the 24‐cycle (i.e. including the night). Conversely, again relative to the growth pattern prior to girdling, growth below the point of girdling would be limited to only the most favourable hours of the day (late night, early morning) using available carbohydrates stored locally (e.g. in bark or xylem ray parenchyma cells, cf., Carlquist 2007). The greenhouse experiment was employed to provide a general test‐bed of model behaviour on an enlarged sample of well‐watered trees for three additional species.

We find that the diel patterns of tree radial growth are variable. In some cases, growth peaks during night‐time hours, when plant water status is at its maximum. In other cases, growth is found to peak at other times of the day or is almost continuous through the 24 h cycle. Our analytical solution (1) isolates irreversible growth from elastic dimensional changes; (2) provides the mechanistic basis for a simple empirical curve‐fitting approach that will abridge future analyses; and (3) helps to clarify the relative roles of sink versus source limitations facilitating the diel cycle of radial growth of trees. A better understanding of the processes driving tree growth is essential to achieve a better predictive capability of the effects of global environmental change on carbon sequestration and water use by forests. Our results highlight the need to consider the diversity of growing strategies across different biomes and tree functional types.

## Theory

Strictly speaking, dendrometers measure the changes in thickness of a tissue at any point in time. Consistent with Mencuccini *et al.* (2013), we use the term ‘thickness’ as opposed to ‘diameter’ when we refer directly to the dendrometer measurements as opposed to the physical tree property. The symbol for thickness is *D*, subscripted *st* for stem thickness, *D*_st_, *b* for bark thickness, *D*_b_, or *x* for xylem thickness, *D*_x_. Thickness changes over time, that is, $\frac{d{\overset{⌢}{D}}_{b}}{dt}$, are the difference of two subsequent thickness readings divided by the corresponding time interval. The caret is employed from now on to describe measured quantities as opposed to modelled ones and differential notation is used to emphasize the near‐instantaneous nature of the processes. A list of variables, meaning and their symbols is given in Table 1. Because xylem thickness can increase or decrease separately from live (or inner) bark thickness (i.e. cambium and phloem), we firstly subtract changes in xylem thickness *D*_x_ from changes in stem thickness *D*_st_ to analyse changes in live bark *D*_b_, that is,

(1) $$\frac{d{\hat{D}}_{b}}{dt}=\frac{d{\hat{D}}_{st}}{dt}-\frac{d{\hat{D}}_{x}}{dt}\text{.}$$

**Table 1:** List of state variables, parameters and their definition for the two models of this paper

Variable/parameter name	Definition	Units
*n*_b_	Number of moles of carbohydrates in the bark	mol
*n*_0_	Number of moles of carbohydrates in the bark at time *t* = 0 (beginning of each measurement)	mol
*t*	Time	s
*F*_gr_	Carbohydrate flux to the cambium	mol s^−1^
*F*_gr,0_	Carbohydrate flux to the cambium at time *t* = 0 (beginning of each measurement)	mol s^−1^
*F*_phl_	Carbohydrate flux from the phloem	mol s^−1^
ε_r,b_	Bark elastic modulus	MPa
*Ψ*_x_	Xylem water potential	MPa
*D*_st_	Stem thickness	m
*D*_x_	Xylem thickness	m
*D*_b_	Bark thickness	m
*D*’_b_	Bark thickness without water content changes	m
*D* ^E^	Elastic component of bark thickness	m
*D* ^P^	Irreversible component of bark thickness	m
*Ψ*_s_	Soil water potential	MPa
*J*_s_	Sap flux density	m^3^ s^−1^
*K*_pl_	Plant hydraulic conductance	m^3^ MPa^−1^ s^−1^
*Π*_b_	Bark osmotic pressure	MPa
*Ψ*_*Π*b_	Bark osmotic potential	MPa
*P*_b_	Bark turgor pressure	MPa
*a*	Rate of flux of carbohydrates per number of moles of carbohydrates in the bark	mol mol^−1^ s^−1^
*R*	Gas constant	m^3^ MPa K^−1^ mol^−1^
*T*	Absolute temperature	° Kelvin
*k*	Rate of xylem and phloem volumetric growth per unit of carbohydrate flux to the cambium	m^3^ mol^−1^
*L*	Radial hydraulic conductance	m^3^ MPa^−1^ s^−1^
*A*	Transverse area between bark and xylem	m^2^
V*_b_	Reference bark volume	m^3^
D*_b_	Reference bark thickness	m
*Δ*	Symbol for quantities zeroed relative to a reference time	—

Changes in live tree bark (from now on, bark) thickness over time comprise irreversible plastic changes (caused by the addition of new wall material on xylem and phloem sides by cellular division and enlargement) and elastic movements (caused by reversible changes in the water content of the bark). The total radial change of the tree bark thickness is defined as the sum of its plastic (irreversible) (*P*) and elastic (reversible) (*E*) components:

(2) $$\frac{d{\hat{D}}_{b}}{dt}=\frac{dD^{P}}{dt}+\frac{dD^{E}}{dt}=\;\frac{dD^{P}}{dt}+\frac{dD_{xyl}^{E}}{dt}+\frac{dD_{osm}^{E}}{dt}$$

where subscript *b* is now dropped in these derived bark‐only quantities for clarity. Plastic changes can only be positive or zero. Elastic changes can be positive, negative or zero, depending on whether the water content of the bark increases, decreases or remains unchanged. The two components of elastic change in bark thickness $\frac{dD_{xyl}^{E}}{dt}$ and $\frac{dD_{osm}^{E}}{dt}$ are caused by two processes, that is, transpiration‐driven changes in xylem pressure potential (i.e. $\frac{dD_{xyl}^{E}}{dt}$) and phloem‐driven and growth‐driven changes in bark osmotic content (i.e. $\frac{dD_{osm}^{E}}{dt}$) (e.g. De Schepper & Steppe 2010). Models predicting $\frac{dD_{xyl}^{E}}{dt}$ already exist (e.g. Zweifel *et al.* 2001; Steppe *et al.* 2006; Hölttä *et al.* 2010). They predict water transfer along a radial gradient of water potential between xylem and bark (e.g. $\frac{dD_{xyl}^{E}}{dt}=\alpha \left(\beta \;{\hat{J}}_{s}-\Delta {\hat{D}}_{b}\right)+\gamma$ Mencuccini *et al.* 2013; Chan *et al.* 2015; Pfautsch *et al.* 2015a). These tension‐driven elastic changes in bark thickness can be estimated from time series of xylem pressure potential Ψ_x_ and bark thickness *D*_b_, relative to an initial reference time. In previous derivations (Mencuccini *et al.* 2013; Chan *et al.* 2015), we employed time series of xylem diameter *D*_x_ to estimate changes in Ψ_x_ using Hooke's law. In other words, in the approach by Mencuccini *et al.* (2013), using the time series of xylem diameter *D*_x_ serves two purposes, that is, it allows for the isolation of bark diameter from stem diameter (cf., Eqn 1) and it allows for the estimation of changes in xylem water potential via Hooke's law. We generalize that approach here, using time series of xylem sap velocity instead of *D*_x_, as a proxy for Ψ_x_ (cf., Supporting Information, Note 1). The equation predicting tension‐driven bark thickness changes resulting from bark water content changes is:

(3) $$\frac{dD_{xy1}^{E}}{dt}=a\left(\beta {\hat{J}}_{s}-\Delta {\hat{D}}_{b}\right)+\gamma$$

where $\frac{dD_{xyl}^{E}}{dt}$ is the predicted tension‐induced change of bark thickness and *Ĵ*_s_ is sap flux density or velocity, a proxy for local (as opposed to leaf) xylem tension. Note that bark thickness is employed as a predictor to derive the tension‐induced changes in bark thickness during the next time interval (Mencuccini *et al.* 2013). It is assumed here that point dendrometers and sap flow sensors are located next to each other on the stem. Parameters *a*, *β* and *γ* have a slightly different definition from Mencuccini *et al.* (2013) because of the incorporation of an additional equation to predict xylem tension using Ohm's law (cf., Supporting Information, Note 1), but the right‐hand side of Eqn 3 remains formally identical to its previous derivation. Assuming that *Ĵ*_s_ is a proxy for xylem tension occurring at the point of *Ĵ*_s_ measurement does not require the assumption of constant soil water potential and soil‐to‐plant hydraulic conductance (cf., Supporting Information, Note 1). However, it does require that the parameters of Eqn 3 are allowed to vary for each day, following the mixed‐model approach outlined in Mencuccini *et al.* (2013). This indirectly allows for the incorporation of the day‐to‐day variability in soil water potential, soil‐to‐plant hydraulic conductance and additional processes, for example, linked to xylem (as opposed to bark) capacitance or potential root/soil disconnections (e.g. Sevanto *et al.* 2008). This approach does not account for short‐term effects resulting from, for example, changes in root pressure (e.g. De Swaef *et al.* 2013b) at time scales of minutes to hours, which are correctly lumped with the osmotic effects described later. Therefore, at the end of step 1 we have:

(4) $$\frac{dD_{b}^{'}}{dt}=\frac{d{\hat{D}}_{b}}{dt}-\frac{dD_{xyl}^{E}}{dt}=\frac{dD^{P}}{dt}+\frac{dD_{osm}^{E}}{dt}\text{.}$$

**Figure 1:**
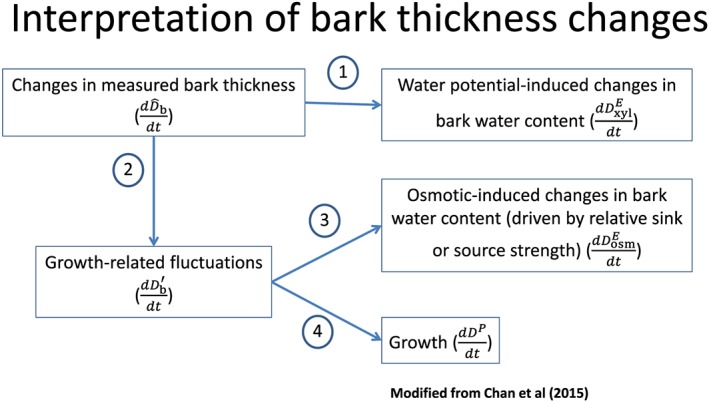
Conceptual diagram explaining the logical sequence of our modelling approach. Changes in observed bark thickness are decomposed into (step 1) an elastic component linked to changes in xylem water potential‐driven bark water use and (step 2) a ‘growth‐related fluctuations’ component, which incorporates irreversible growth plus further elastic changes because of transient osmotic imbalances. Once the ‘growth‐related fluctuations’ are obtained, Eqn 6 is employed to separate the elastic changes because of transient osmotic imbalances (step 3) from irreversible growth (step 4). Variable symbols are explained in Table 1.

**Figure 2:**
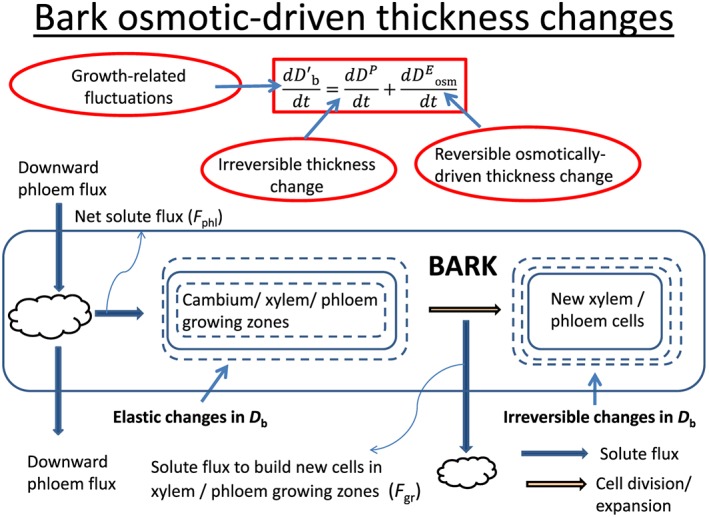
Conceptual diagram employed to separate the ‘growth‐related fluctuations’ term into the (osmotically driven) elastic term and the irreversible growth term. Elastic and irreversible diameters, *D*, are abbreviated using the superscript letters ‘*E*’ and ‘*P*’, respectively, while the subscript letters ‘b’ stands for bark and ‘osm’ for osmotically driven, respectively. The model is based on a mass balance analysis with one‐compartment (the bark), one inflow (*F*_phl_) from the phloem supply region and one outflow (*F*_gr_) towards the xylem/phloem growing zones. The only state variable is *n*_b_, the number of moles of carbohydrate in the bark. The amount of carbohydrates in the phloem supply region is not represented explicitly in the model (cloud on left‐hand side of graph). The orange horizontal arrow in the centre represents the transition from the growing zone (in which elastic movements occur) to the mature xylem/phloem cells (where growth is irreversible). The vertical blue arrow to a second cloud at the bottom represents the flux of carbohydrates resulting from the production of new cells, which is associated with the orange arrow directly above.

$\frac{dD_{b}^{'}}{dt}$ are the estimated changes in bark thickness after the tension‐driven changes in bark water content have been subtracted. In Chan *et al.* (2015), $\frac{dD_{b}^{'}}{dt}$ was referred to as ‘growth components curve’. Because $\frac{dD_{b}^{'}}{dt}$ combines an element of elastic change with an element of irreversible change, we refer to it here instead as ‘growth‐related fluctuations’. A second model is now employed to predict whether changes in bark osmotic content result in bark water content changes. Bark osmotic content changes are caused by differences in the balance between solute supply and demand (cf., Fig. 1 for a pictorial description of this dual approach).The model describing osmotically driven elastic thickness changes of the bark is obtained as follows. In the Supporting Information (Note 2), we employ a simplified mechanistic model to show that, over the time scale of typical dendrometer measurements (i.e. tens of minutes), irreversible growth and osmotically driven elastic changes are linearly related to each other, that is,

(5) $$\frac{dD^{P}}{dt}=m+r\frac{dD_{osm}^{E}}{dt}$$

where *m* and *r* are two parameters estimated from the data and $\frac{dD^{P}}{dt}$ and $\frac{dD_{osm}^{E}}{dt}$ refer to irreversible growth and osmotically driven elastic movements, respectively. A direct relationship between the component of elastic expansion/contraction driven by bark osmotic content changes and irreversible thickness changes is expected from mass balance arguments, because any imbalance between carbon supply and demand will result in a net change in bark solute content (e.g. Hölttä *et al.* 2010; De Schepper & Steppe 2010). Osmotically driven net shrinkage and swelling may then temporarily occur in the bark, unless a perfect osmotic steady state exists between inflows and outflows. This transient imbalance may be caused by a source limitation (i.e. supply lower than current consumption by growth in relative terms, i.e. bark osmotic content declines and volume shrinks) or by a sink limitation (i.e. net growth lower than supply in relative terms, i.e. carbohydrates accumulate and bark swells). The main processes, fluxes and state variables are summarized in Fig. 2. Parameters *m* and *r* have a physical interpretation:

$$m=\;\frac{k}{A}F_{gr,0}\;\text{and}\;r=\frac{\varepsilon_{r,b}\;k}{RT}l\text{.}$$

*Here, k* is the combined phloem plus xylem volumetric growth per unit of carbohydrate supply, *A* the vertical area of contact between cambium and growing zone, *ε*_r,b_ the bark radial elastic modulus, F_gr,0_ the rate of phloem carbohydrate supply to the cambium at the beginning of the time interval of measurements, *R* the gas constant, *T* temperature and *l* a scalar constant dependent on the time interval between measurements. Equations 4 and 5 can be solved simultaneously to eliminate $\frac{dD_{osm}^{E}}{dt}$ to give:

(6) $$\frac{dD^{P}}{dt}=\frac{m}{1+r}+\frac{r\;}{1+r}\frac{dD_{b}^{'}}{dt}\text{.}$$

Equation 6 defines an inverse problem that can be solved to predict the irreversible changes in bark thickness $\frac{dD^{P}}{dt}$ associated with changes in growth‐related fluctuations $\frac{dD_{b}^{'}}{dt}$ using statistical minimization techniques. In other words, the time series of bark thicknesses (corrected for tension‐driven water content changes) is employed to derive the underlying time series of irreversible radial growth. The differential notation here should be interpreted as referring to finite differences over the time scale during which measurements are carried out. Because the system is linear, no errors are made by moving from differentials to finite differences over 10 to 30 min intervals. In essence, one minimizes the differences between $\frac{dD_{b}^{'}}{dt}$ and predicted irreversible changes $\frac{dD^{P}}{dt}$ by applying Eqn 6, subject to biologically sensible constraints, that is, $\frac{dD^{P}}{dt}\geq 0$ and $D_{b}^{'}\geq D^{P}$ always, that is, osmotically driven elastic expansion/contraction cannot include the changes in irreversible diameter growth underneath. Overall, to identify the mean diurnal patterns of tree growth, the dual approach outlined here requires the estimation of five parameters, *a*, *b*, *γ*, *m* and *r*. However, if the main interest resides in interpolating seasonal time series and within‐day patterns are not of interest, three parameters need to be estimated, that is, *a*, *b* and *γ*, as will become clear after the Results section. In the approach outlined by Mencuccini *et al.* (2013), two time‐series are required, stem and xylem thickness changes, respectively. In the approach outlined here, a third time‐series is required, that is, xylem sap velocity (or sap flux density), which is employed to derive an indirect estimate of xylem water potential, while xylem thickness changes are only used to separate stem from bark thickness changes.

## Materials and Methods

### Experimental setup

To determine the mean daily patterns of radial stem growth, we selected species from a range of environments and functional types (i.e. angiosperms and gymnosperms, deciduous and evergreen). We selected two experimental manipulations which would allow testing the proposed model. For each species, we restricted our analyses to periods when active growth was taking place.

1. Irrigation experiment

This study was performed in a Scots pine stand in the Rhone valley in Switzerland (Pfynwald, 46° 18′ N, 7° 36′ E, 615 m a.s.l.), one of the driest inner valleys of the European Alps (mean annual temperature: 9.2 °C, annual precipitation sum: 657 mm, Bigler *et al.* 2006) on an alluvial fan and debris cone of the Ill river. The soil was a shallow and skeletal para‐rendzina (soil depth 60 cm) characterized by low water retention. The forest, while dominated by Scots pine, included occasional *Quercus pubescens* Willd. individuals (Brunner *et al.* 2009). Scots pines were on average 100 years old with a height of 10.8 m, stem density of 730 stems/ha and basal area of 27.3 m^2^/ha. The experimental site comprised eight plots, of which four plots were irrigated from April to October every year since 2003 with an additional 700 mm/year of water and four plots remained non‐irrigated and acted as control treatment (see Dobbertin *et al.* 2010, for more details). Air and soil temperature, air humidity, PAR, wind speed, precipitation and soil water potential were recorded on a weather station (Campbell Sci. Inc., Logan, UT, USA). VPD was calculated from air humidity and temperature. In April 2013, three trees were selected in one control plot and one irrigated plot. Selected trees were representative of average tree health, based on visual estimation of canopy defoliation. Sap flux density was measured using thermal dissipation sensors (Granier 1985) inserted into the xylem at breast height. The sensors were covered by aluminium trays and reflective bubble wrap to minimize natural temperature gradients. Two sensors were installed on each tree on the East‐ and West‐facing sides of the trunk. Calculations of sap flux density in the outer and central sapwood and determination of zero flow conditions followed Granier (1985) and Poyatos *et al.* (2013). A pair of point‐dendrometers (LVDT, DG/2.5, Solartron metrology, Bognor Regis, UK) was used to measure thickness variations of xylem and living bark. Both sensors were installed on a rectangular frame made of out of invar (Sevanto *et al.* 2005). Temperature of the invar frame and the trunk (below dead bark) was monitored with thermocouples to correct for thermal deformation of frame and wood (Martínez‐Vilalta *et al.* 2007). The head of the LVDT, measuring changes in xylem diameter rested on the head of a screw inserted approximately 10 mm into the stem sapwood. The head of the second LVDT, measuring whole stem radius, rested directly on the living bark. The living bark had been previously exposed by opening a 1 cm side window in the dead bark with a surgical knife and was coated with silicone grease to avoid desiccation. Data were recorded every 10 s before being averaged on a 30 min basis.

- Girdling experiment

Twelve whole‐tree chambers equipped with temperature control to track ambient conditions and capable of measuring whole‐tree net carbon and water exchange were deployed at the Hawkesbury Institute for the Environment (Richmond, Australia). Climate at the site is subtropical, mean annual temperature is 17 °C and mean annual precipitation is around 800 mm. *E. tereticornis* trees were grown in pots for three months before being transplanted into the chambers. One tree was grown in each of six chambers for 17 months (December 2012 – May 2014). The trees were subjected to an experiment involving two levels of controlled soil watering (‘control’ and ‘dry‐down’, i.e. three trees for each watering level). Controlled irrigation occurred on average every 14 days with an amount of water equal to half the mean monthly rainfall. In the ‘dry‐down’ treatment, irrigation was stopped from 19 January until 2 May when all trees received again equal amounts of water. Watering was repeated on three more days (3–5 May) on the previously mildly droughted trees. Periodic monitoring of leaf water potential conducted throughout the experiment indicated that trees had fully rehydrated two weeks after the end of the dry‐down (Pfautsch et al., in preparation). On 16 May 2015, all six trees were stem‐girdled. To carry out the girdling, a 5‐cm‐wide collar of bark was removed from the stem 0.7 m above ground. The exposed sapwood was wrapped in wet paper towels and plastic to prevent dehydration. Occurrence of new growth in the exposed windows was periodically checked. Paper towels were rewetted daily. At the time of girdling, total tree height ranged from 8.02 to 9.34 m and basal stem diameter from 78 to 103 mm. Ten days after girdling (26–27 May 2015), all trees were manually defoliated, and subsequently felled and removed from the chambers. The full experiment involved a temperature treatment crossed with the watering treatment on six additional trees, but those trees were not instrumented with sap flow sensors and stem dendrometers. A full description of the experimental setup is given by Barton *et al.* (2010). Whole‐tree gas exchange fluxes for the control and the high‐temperature treatments were reported by Drake *et al.* (2016). Gas exchange fluxes for the watering and girdling treatment will be reported in a separate publication (Pfautsch et al., in preparation).

Two sap flow sensors (Edwards Industries, New Zealand) were installed along the main stem on each of the six trees. One sensor was positioned below the canopy (‘bottom’ position, average of 0.6 m height), the other inside the upper tree canopy (‘top’ position, average of 3.3 m height). With regard to the girdling experiment, we will refer to the bottom installation as ‘below’ the point of girdling, and the one further up the stem as ‘above’ girdling. Twenty‐four high‐resolution point potentiometers (ZN11‐O‐WP, Zweifel Consulting, Hombrechtikon, Switzerland; cf., Zweifel *et al.* (2014)) were also installed in pairs next to the sap flow sensors mounted on circular carbon‐fibre frames following Pfautsch *et al.* (2015b). The dendrometer setup at each height consisted of two dendrometers, one installed over the bark region and the second directly on the xylem, in a small area where a window of bark had been cut and the xylem directly exposed. To avoid dehydration of the xylem, the area surrounding the tip of the dendrometers on the xylem was covered with silicon grease. All gas exchange and sap flow systems logged data at 15 min intervals. Dendrometer data were logged at 5 min intervals but averaged to 15 min (CR1000, Campbell Scientific, Logan, USA). Our analysis covers primarily the portion of the experiment related to the pre‐ and post‐girdling period.

- Greenhouse observations

Measurements of stem and xylem thickness changes were also carried out on three well‐watered trees belonging to three species, *Eucalyptus globulus* (Labill.), *Pinus radiata* (D. Don) and *Alnus jorullensis* (Kunth). The three trees (one per species) used for this component of the study were 2–3 years old, growing in a glasshouse in Hobart, Tasmania (42.88° S, 147.32° E). At the start of the experiment, the trees were 2–3 m tall and were grown in a potting mix consisting of eight parts composted pine bark to three parts coarse river sand. The potting medium contained low phosphorus premium controlled‐release fertilizer (N:P:K—17.9:0.8:7.3). The plants were kept well‐watered throughout the experiment, which lasted for approximately two weeks. Changes in stem thickness at the bark surface and at the sapwood/xylem surface were measured side by side on the stem at 0.1–0.2 m above the soil surface. Contact on the sapwood tissues was achieved by removing a small portion (ca 10 mm circle) of bark and periderm. The radial dendrometer consisted of two high resolution differential variable reluctance transducers (HSG‐DVRT‐6, resolution ± 0.6 *μ*m, Microstrain, Williston, USA) mounted on an alloy bracket 35 mm apart, attached to the stem via a 2.3‐mm‐diameter alloy rod and backing plate. The signal from each transducer was conditioned (DEMOD‐DC2, Microstrain, Williston, USA) and recorded on a data logger every 15 min (CR 1000, Campbell Scientific, Logan USA). Sap flow was measured using the heat ratio technique (Burgess *et al.* 2001) using one sensor per tree logged every 15 min (SFM‐1, ICT International, Armidale, Australia). Sensor probes were installed at c.a. 0.2 m above the soil surface and inserted so that heat pulse velocities were measured at a depth of 5 mm below the bark/sapwood boundary.

### Statistical analyses

#### Tension‐driven bark water use model

All statistical analyses were carried out in R 3.0.2 (R Core Team 2014). Linear modelling of the relationship depicted in Eqn 3 was carried out using the nlme library (Pinheiro & Bates 2000), following Mencuccini *et al.* (2013), allowing Eqn 3 to be fitted using standard regression techniques. Employing Eqn 3 to predict xylem‐tension driven changes in bark thickness $\frac{dD_{xyl}^{E}}{dt}$ based on measured bark thickness ${\hat{D}}_{b}$ and xylem sap velocity *Ĵ*_s_ is straightforward in the case of the Tasmanian greenhouse monitoring, where individual plants did not belong to any treatment. Its application to the Australian field girdling and the Swiss irrigation experiments needed to consider that plants were nested within experimental treatments, for which the parameters of Eqn 3 could vary accordingly. For the Australian field girdling experiment with *E. tereticornis*, Eqn 3 was therefore modified to include the following independent variables as fixed factors: bark thickness, sap velocity, watering treatment (control/dry‐down), sensor position (base/top) and the interactions between these last two variables and bark thickness or sap velocity. This allowed making the parameters of Eqn 3 dependent on the two watering levels and to account for the two positions of sap velocity and dendrometry sensors. An autoregressive correlation structure (*p* = 1) was employed to allow for lack‐of‐independence over time among measurements for each day. Testing of significance of fixed effects, random effects and autocorrelation structure followed Mencuccini *et al.* (2013). The final model, selected on the basis of its *Δ*AIC relative to competing models, employed day as the random factor, and the fixed components included only bark thickness, sap velocity and the interaction term between bark thickness and sensor position (base/top). Notably, the watering treatment was not even marginally significant (*P* > > 0.05) and was excluded from the final model. We firstly derived the parameters of Eqn 3 using bark thickness and sap velocity data from before the experimental girdling at both stem heights separately. We then applied these estimated parameters and Eqn 3 to the bark thickness and sap velocity data after the girdling for both stem heights separately. For the Swiss irrigation setup, we employed a similar model structure for Eqn 3, whereby the fixed component of the model included the irrigation/control comparison, bark thickness and sap velocity. In this case, the parameters were derived separately for each treatment, as treatment was a significant variable in the model. The R code employed for these statistical analyses, in addition to the code employed to implement Eqn 3, is included in Note 3 of the Supporting Information.

#### Osmotic‐driven growth model

To separate irreversible growth from osmotically driven elastic changes in bark thickness, we employed a global optimization technique subject to constraints based on Eqn 6 on all species. Coefficients *m* and *r* in Eqn 6 can vary throughout the day and from day to day reflecting variable conditions of carbohydrate supply and demand. Their mean diurnal behaviour can be estimated given a long enough time series (cf., Discussion in Supplementary Note 2, for further details). Because of its ability to locate global minima inside constraint boxes, we employed a modified Differential Evolution (DE) algorithm (library DEoptimR, Brest *et al.* 2006; Zhang & Rangaiah 2012). To simplify estimation, *m* and *r* were kept constant for consecutive intervals of four hours during each day (early morning—5–8 am, late morning—9–12 pm, afternoon—13–16 pm, evening—17–20 pm, early night—21–24 pm and late night 1–4 am) but were allowed to vary across the six daily periods and across days. Initial values of the parameters were obtained by drawing values uniformly within pre‐specified boundaries (Mullen *et al.* 2011). We ran our analysis by varying the number of hours (from two to eight) over which *m* and *n* were kept constant. Our conclusions about daily trends in $\frac{dD^{P}}{dt}$ are robust to these changes, the only difference being in the number of iterations required to achieve convergence (the larger the parameter space, the higher the number of iterations). Using intervals of four hours and a tolerance of 1 × 10^−9^, the DE algorithm converged in between 1 and 6 h on a quad‐core laptop, depending on time series length. Following convergence, estimates of plastic and osmotically driven elastic thickness changes, as well as parameter estimates were obtained for each time interval and for each day in the time series (cf., Supplementary Figs S1–S2 for examples of function and parameter convergence during the search procedure). The R code employed to implement the osmotic‐driven growth model is included in Note 4 of the Supporting Information.

Tests of significance of diel patterns of diameter growth were done by fitting a generalized additive mixed model using the gamm4 library. We employed tensor product smooth terms calculated separately for each treatment (irrigation versus control in Switzerland; control versus dry down before girdling and, after girdling, above‐girdling versus below‐girdling in Australia) to model the relationship between diameter growth and time of day, with treatment as a fixed factor. To test for differences because of overall growth rates between treatments, we tested the significance of the coefficient for the fixed factor (i.e. intercept shift). To test for difference because of the daily rhythm between treatments, we tested whether the model employing the smooth terms calculated separately for each treatment gave a significantly better fit compared to the model with smooth terms calculated jointly for all data using a one‐degree *χ* ^2^ test.

## Results

**Figure 3:**
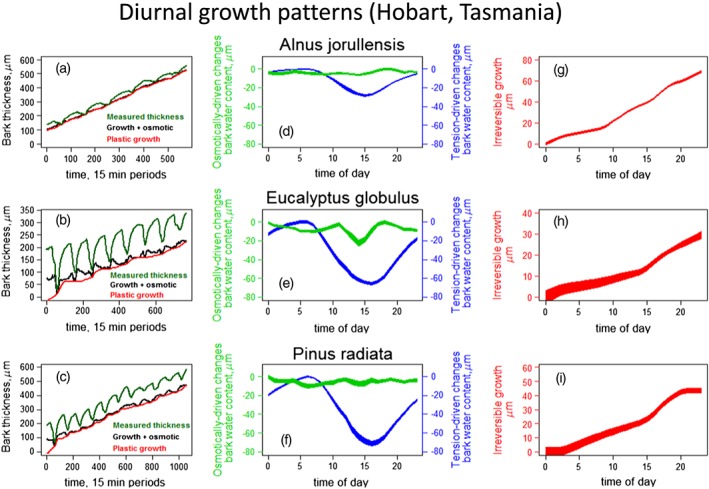
Three example solutions of the application of the two models based on Eqns 3 and 6. Left panels, a to c) Application of the coupled models for three different species (*Alnus jorullensis, Eucalyptus globulus, Pinus radiata*) in a greenhouse in Hobart, Tasmania. Green curve, raw bark thickness readings from the point dendrometers; black curve, ‘growth‐related fluctuations’ curve from which water potential‐driven (for brevity, tension‐driven) elastic changes have been subtracted; red curve, plastic irreversible growth. Central panels, d to f) Averages across the sampled period of the 24 h cycles of the two elastic curves, that is, tension‐driven bark water content changes (blue curve), and osmotically driven bark water content changes (green curve). The blue and green bands give one standard error around the mean (not shown). Right panels, g to i) Averages across the sampled period of the 24 h cycles of the irreversible stem radial growth. The red bands give one standard error around the mean (not shown). Note the different scales for the three species.

An exemplary application of our dual‐approach analysis is presented in Fig. 3 (panels a to c) for three species, *A. jorullensis*, *P. radiata* and *E. globulus*, grown under identical environmental conditions in a greenhouse in Tasmania. In all three cases, measured bark thicknesses (panels a to c, green curves) showed daily fluctuations larger or equivalent in magnitude to the actual growth signal, making the isolation of intra‐day and across‐days growth problematic. In all three cases, the majority of the intra‐day fluctuation was accounted for by the dynamics of the transpiration‐driven changes in bark water storage, as predicted by Eqn 3. Subtracting the transpiration induced changes in bark thickness from the raw bark thickness reading gives the ‘growth‐related fluctuations’ curve (black curve). The osmotic model based on Eqn 6 predicted additional elastic fluctuations in bark water content caused by changes in bark osmotic content (difference between black and red curves), although these changes were much smaller compared to the first ones. Once this second signal was subtracted from the ‘growth component’ curve, irreversible diameter growth could be isolated (red curves). From Fig. 3, it is apparent that the red line for predicted irreversible growth always approximates the daily minima of the black curve. Interpretation of the behaviour of the curve is facilitated by examining the model. Setting $\frac{dD^{P}}{dt}=0$ in Eqn S20 shows that elastic changes at times of no plastic growth can either be positive, negative or zero depending on a critical value of parameter *a* (cf., red and black curves, Fig. 3 left panel for *E. globulus*, for one example). Conversely, setting $\frac{dD_{osm}^{E}}{dt}=0$ in Eqn S19 shows that bark osmotic content at the time of the daily minimum in the black ‘growth‐related fluctuations’ curve can vary depending on concurrent plastic growth, *a* and *k* (cf., same panel of Fig. 3 as above).

Mean daily time courses of changes in bark thickness caused by the two processes mentioned above (xylem tension‐driven and bark‐osmotic content‐driven changes in bark water content) are directly compared in Fig. 3 (panels d to f). The diurnal cycle of tension‐driven changes in bark water content is common to the three species, with almost identical periods (albeit with varying magnitude) across species. In contrast, the diurnal cycle of bark water content driven by changes in bark osmotic content is variable. While one species (*E. globulus*) showed a clear periodicity, with minima found in the early afternoon, seedlings of the other two species showed relatively little diurnal dynamics. By way of comparison, for the Scots pine experiment in Switzerland, the diurnal cycle of bark water content driven by changes in bark osmotic content was very pronounced in the control (severely droughted) trees, but much smaller in the irrigated trees (Supporting Information, Fig S3). In the *E. tereticornis* experiment in Australia instead, the diurnal cycle of bark water content driven by changes in bark osmotic content was generally very small, similar to the observations made in the Tasmanian experiment (Supporting Information, Figs S3 and S4).

Mean daily patterns of irreversible growth for the three trees in the Tasmanian experiment are also given in Fig. 3 (panels g to i). In *A. jorullensis*, radial growth was almost continuous through the 24 h cycle. *E. globulus* showed a similar trend, whereas in *P. radiata* growth slowed down during the night.

**Figure 4:**
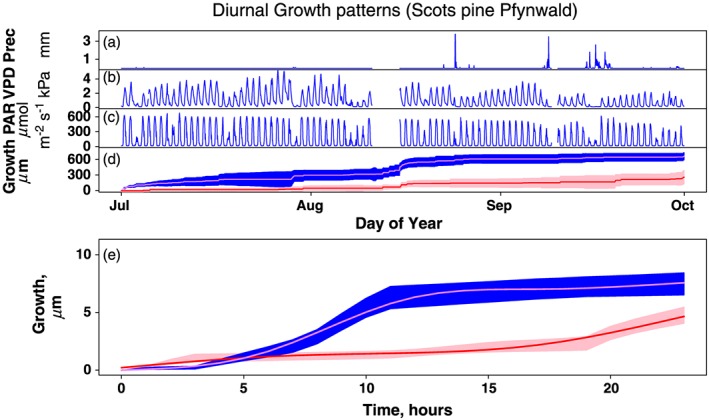
Test of model performance in a field irrigation experiment on Scots pine at Pfynwald, Switzerland. Panels a to c at the top provide environmental information for the summer of 2013 (early July through to end of September), which coincided with the main period of radial growth at this site. Top panels represent traces of precipitation events Precip (panel a), air vapour pressure deficit VPD (panel b), photosynthetically active radiation PAR (panel c) and mean seasonal growth rates for trees in the control (pink) and the irrigated (blue) plots (panel d). Panel e gives the mean inferred daily pattern of irreversible growth for the control trees (pink) and the irrigated trees (blue). For both panels, the central lines within each band give the mean curves. Control trees grew more slowly than irrigated trees overall and their daily rhythm of growth was largely limited to night. Differences in absolute rates of growth and the daily rhythms of growth between control and irrigated trees were highly significant (both *P* < 0.001).

Finally, under two experimental conditions (i.e. the irrigation study in Switzerland and the girdling study in Australia), we tested whether our model predicted diel patterns of diameter growth consistent with theoretical predictions. The mean within‐day patterns of thickness increase of Scots pine in a field irrigation experiment in Switzerland are shown in Fig. 4. The final mean daily thickness increment was in the same order of magnitude across plots of the two treatments, albeit significantly larger (gamm intercept for treatment, *P* < 0.01) for the irrigated compared to the control plot (Fig. 4e). However, the within‐day distribution of increases in thickness differed significantly between the two plots (gamm difference for smooth terms, *P* < 0.01), with faster rates of growth during daytime in the irrigated plot, compared to a pattern of growth primarily occurring at night in the control plot.

**Figure 5:**
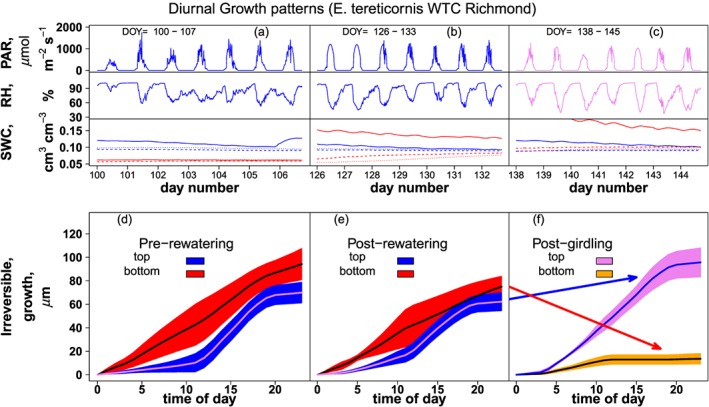
Test of model performance in a field stem‐girdling experiment on *Eucalyptus tereticornis* at Richmond, New South Wales, Australia. Panels at the top provide environmental information for three periods throughout the summer of 2014 (days of year 100 to 107, 126–133 and 138–145). Panels represent traces of photosynthetically active radiation PAR (panels a–c top), air relative humidity RH (panels a–c middle) and soil relative water content SWC (panels a–c bottom) (control trees in blue; partially droughted and rewatered trees in red; different line types represent different soil depths). The first period represents the initial phase of the experiment, when three trees were mildly droughted and three were continuously watered. The second period represents the second phase of the experiment when the mildly droughted trees were re‐watered. The third period represents the post‐girdling phase, starting three days after the girdling had been carried out. The bottom panels d–f give the mean inferred daily patterns of growth, for two positions along the stem and the same three time periods. Growth for the top position is given in blue and for the bottom position in red. The ‘top’ stem position corresponds to a point above girdling; the ‘bottom’ position corresponds to a point below girdling. No differences were found between the mildly droughted and the control trees for either position and for either period (cf., text for more information); therefore, their values were averaged, and data are presented only as a function of position along the stem. The colour scheme in panel f has been changed to highlight the post‐girdling relative to the pre‐girdling period in d and e. Arrows link pre‐ and post‐girdling colour scheme.

Within‐day patterns of thickness increase of *E. tereticornis* before and after girdling in the Australian experiment are shown in Fig. 5. Prior to girdling, no significant difference was found between the two controlled watering regimes (gamm effect for treatment, *P* > 0.05, for both stem positions and both prior to and after re‐watering, cf., Supporting Information, Fig. S5); therefore, data were pooled across watering levels. The two positions showed significantly different diel rhythms prior to girdling both prior to and after re‐watering (Fig. 5, gamm effect for smooth terms, both *P* < 0.001), but no difference in absolute rates of growth (i.e. for their intercept term, *P* = 0.082; *R* ^2^ = 0.70, and *P* = 0.20; *R* ^2^ = 0.70). The diel patterns dramatically diverged after girdling (gamm effect for treatment and smooth terms, both *P* < 1 e^−8^; *R* ^2^ = 0.87). Growth above the girdling point increased in magnitude, while growth below the girdling point decreased (both differences in absolute growth rates *P* < 0.01; *R* ^2^ = 0.71 and 0.87, respectively). In addition, below the point of girdling, thickness increases were close to linear prior to girdling in both periods; however, they only occurred for a few hours at the end of the night and in early morning after girdling (gamm effect for smooth terms, *P* < 1 e^−15^). In contrast, thickness increases above the point of girdling peaked during afternoon hours prior to girdling, whereas thickness increases continued almost throughout the 24 h cycle except the early and middle parts of the night after the girdling (gamm effect for smooth terms, *P* < 1 e^−15^). Environmental conditions remained relatively comparable throughout the three periods.

## Discussion

This paper reports the first analytical solution for the isolation of stem diameter growth of trees from continuous field readings of stem dendrometers. Klepper *et al.* (1971) were the first to employ modern LVDT sensors to continuously and precisely monitor stem growth at high temporal resolution. In their discussion, they noted the occurrence of hysteresis loops in the relationship between leaf water potentials and stem diameters and concluded that stem diameter changes reflect changes in stem tissue hydration. Eliminating transitory capacitance from irreversible growth has proven difficult. Numerical models predicting growth based on the Lockhart equation (e.g. Génard *et al.* 2001; Steppe *et al.* 2006) or on combinations of the Lockhart equation plus phloem sugar transport (Hölttä *et al.* 2010; De Schepper & Steppe 2010) already exist. However, the present contribution is unique in that the model (1) represents and distinguishes the xylem tension‐driven and osmotic content‐driven processes involved in stem thickness changes without implying any mechanism controlling radial growth, and (2) the five parameters (and associated uncertainty) can be derived directly from statistical fits (i.e. by fitting the models to the data). None of the parameters derived here are taken from literature values and for all of them the uncertainty can be quantified.

The theoretical demonstration we present combines two models in series. In the first step, a mixed‐effects model is applied to derive a function that separates bark hydraulic storage from ‘growth‐related fluctuations’. The ‘growth‐related fluctuations’ curve (growth plus osmotic changes) is the predicted bark thickness change after accounting for changes in xylem tension. In the second step, a constrained optimization routine is employed to fit the theoretical equation for irreversible growth to the ‘growth‐related fluctuations’ curve to separate out the residual fraction of variation attributable to osmotically driven elastic changes. Importantly, the optimization routine minimizes the difference between ‘growth‐related fluctuations’ and predicted irreversible growth. In other words, it determines the minimum amount of osmotic‐induced elastic changes that need to be invoked to predict zero or positive irreversible growth, given the time series and the assumed osmotic model. While this approach is useful in providing a mean within‐day pattern of irreversible growth, it does not need to be applied at the seasonal time scale, if the main interests are the long‐term growth patterns and not the within‐day variations. It follows from the minimization approach that a simple linear interpolation between the daily minima of the ‘growth‐related fluctuations’ curve effectively gives the seasonal curve for irreversible growth, at the cost of being unable to determine within‐day patterns accurately. This empirical interpolation considerably simplifies the problem of isolating irreversible growth. Isolating growth at a seasonal time scale requires (1) applying Eqn 1 of this text to separate bark from xylem thickness fluctuations; (2) applying the Mencuccini *et al.* (2013) model or Eqn 3 of this text to the raw bark thickness and sap velocity data to derive the tension‐induced changes in bark thickness; (3) subtracting these predicted changes because of xylem tension from the raw bark thickness data; and (4) interpolating the daily minima of the obtained curve.

The osmotically driven model presented here is based on a solution to the problem of growth as a balance between relative carbon source strength and carbon sink demand. The model cannot quantify source and sink strength in an absolute sense and it has therefore a reduced scope compared to models directly addressing the question of the links between source strength of photosynthesis and sink strength of stem radial growth (e.g. the CASSIA model, Schiestl‐Aalto *et al.* 2015). However, it can attribute the observed cycles of shrinkage or swelling of the ‘growth‐related fluctuations’ curve to changes in the relative magnitude of source and sink strength. A ‘growth‐related fluctuations’ curve increasing at a constant rate (i.e. a straight line with positive slope) implies a balance between source and sink strength. Transient swellings in the curve imply that the source is relatively stronger than the sink. Conversely, transient shrinkages imply that the source is relatively weaker than the sink. This is perhaps most easily seen in the daily patterns for the control (severely droughted) Scots pine trees in Pfynwald (Fig. S3, top right panel), where the night‐time and early‐morning osmotically induced swelling of the bark corresponds to periods of active growth (cf., Fig. 4, bottom panel), suggesting that growth is occurring here at a time when the bark is replete with carbohydrates and turgor conditions are favourable. Parameter *m* of Eqn 4 provides information on the rates of irreversible growth at different times of the day (the product *kF*_gr,0_ gives the irreversible thickness growth for a given flux of carbohydrate to the bark), while parameter *r* relates to the interplay between irreversible growth and elastic properties affecting the transients. The approach is based on two assumptions (cf., Supporting Information Note 2, for a complete discussion), (1) that the carbohydrate flux to the growing cells depends as a first‐order process on bark carbohydrate content and (2) that the rate of cell division/enlargement is proportional to phloem carbohydrate flux to growing cells. The first assumption could be relaxed by employing Michael–Menten kinetics; however, this is highly unlikely to change the conclusions, which are dependent on the occurrence of the process rather than the nature of a linear vs. curvilinear response. The second assumption forms the basis of several other models of plant growth (e.g. Hölttä *et al.* 2010; De Schepper & Steppe 2010; De Swaef *et al.* 2013a) and reflects our understanding that growth and carbohydrate supply must be coupled (e.g. Tardieu *et al.* 2012; De Swaef *et al.* 2015; Steppe *et al.* 2015; Deslauriers *et al.* 2016). The osmotic content‐driven model makes no assumption about causal processes limiting growth, that is, whether growth is primarily controlled by turgor at the sink, hydraulics or by supply limitations. Because the model allows the two parameters *m* and *r* to vary as a function of time of day, the inferred diel patterns of growth only depend on the shape of the original dendrometry data (stem and xylem) and the daily shape of the sap flow data.

We employed two experiments to test the logic of our modelling approach. In the Scots pine case study, we hypothesized that radial growth would be more concentrated during night‐time in the control (droughted) plot, but would occur also during day‐time in the irrigated plot. In the *Eucalyptus* case study, we hypothesized that day‐time radial growth would be more limited by girdling below the point of girdling, whereas the accumulation of photosynthetic products above the point of girdling would result in a growth pattern more evenly distributed during day‐time and night‐time periods. Both of these predictions are reflected in our data. The variability in the daily rhythm of radial stem growth that we found across the five case studies was expected. Although simple theory would anticipate a control entirely based on diurnal changes in plant water potential, much of the literature highlights instead the complexity of the processes involved in leaf and root growth (e.g. Tang & Boyer 2002; Tardieu *et al.* 2012). We surveyed the literature for past reports on the daily rhythm of primary height growth (stem leader) of trees, a variable much less affected than tree diameter by periodic changes in water content of the stem (Table 2). Equally variable patterns of axial extension growth have been reported in the field, with many studies showing growth peaks in the afternoon, rather than night or morning hours. These results are inconsistent with the theory that radial stem growth is uniquely determined by daily patterns of water potential, a conclusion that is also prevalent in many of the articles cited in Table 2. Although many of the studies reported in Table 2 highlight night‐time temperature as an additional variable affecting growth, it is likely that several other variables may have changed during the daily cycle. For example, carbohydrate availability in the bark is a strong possibility, with its indirect effect on the availability of substrates for growth and of osmotic solutes for turgor regulation (e.g. De Swaef *et al.* 2013a and 2015; Deslauriers *et al.* 2016).

**Table 2:** Summary of available literature on diurnal patterns of stem leader growth in forest trees. For each reference, the species, the periods of maximum and minimum growth, the period of observation and the analysis of the factors affecting the growth pattern are given. Notes: T, temperature; WP = plant water potential

Species	Period of maximum growth	Period of minimum growth	Period of observation	Interpretation	Author
*Eucalyptus regnans*	Variable: often mid afternoon	Variable: often early morning/nighttime	December	T interacted with WP changes	Cremer 1976
*Picea abies*	Afternoon	Early morning	June	—	Hertz 1929
	Mid‐late morning Late afternoon Late afternoon	Nighttime Early morning Early morning	June	T interacted with WP — T interacted with WP	Odin 1972; Mork 1941; Worrall 1973
*Picea sitchensis*	Late afternoon/ Evening	Morning/midday	July	T interacted with WP changes	Milne *et al.* 1977, 1983
*Pinus contorta*	daytime	Nighttime	—	T interacted with WP changes	Worrall 1973
*Pinus palustris*	nighttime	Daytime	April	—	Gilbert *et al.* 2006
*Prunus persica*	afternoon	Nighttime in March–Apr; early morning in May–Jun	May to June	T interacted with WP changes	Basile *et al.* 2003
*Pinus radiata*	Late afternoon (constant rate during rainy days)	Morning (constant rate during rainy days)	October	T interacted with WP changes	Cremer 1976
*Pinus resinosa*	nighttime	Daytime	June	—	Keinholz 1934
*Pinus sylvestris*	Afternoon	Early morning	June	WP interacted with 3 h delay T changes	Hertz 1929; Kanninen 1985
*Zinnia elegans*	Early morning	Late afternoon	—	—	Neily *et al.* 2000

The differences between the approach presented by Zweifel *et al.* (2016) and the one developed here must be noted. In the case of Zweifel *et al.* (2016), an empirical interpolation was conducted across the maxima of the raw stem diameter readings. Here, the ‘growth‐related fluctuations’ curve is extracted from bark thickness data, and then the minima of this curve were interpolated. While not identical, it is possible that, under certain conditions, the two approaches provide similar answers to the question of isolating growth. The present approach also provides information on bark water use and should help inform source–sink carbon balance analyses and the analysis of the coupling between canopy photosynthesis, stored carbohydrate reserves and wood growth.

## Conclusions

We present an analytical method that allows the complete separation of the daily shrinkage/swelling patterns of stem dendrometer data into components of elastic change in bark thickness and irreversible radial growth. Our conclusion that daily patterns of growth may vary from case study to case study is consistent with the hypotheses tested in two field experiments (irrigation and girdling) and with much of the available empirical evidence of stem leader growth of trees. The application of the osmotic content‐driven model derived here is not necessary at the seasonal time scale, because a linear interpolation of the minima of the regression function following Mencuccini *et al.* (2013) suffices to isolate irreversible growth. Further work is necessary to determine how this method compares with more empirical approaches that document stem diameter growth at high temporal resolution.

## Supporting information


**Note 1.** Details of the bark water use model
**Note 2.** Details of the osmotic model.
**Figure S1.** Typical example of convergence of the function employed to derive irreversible growth from the ‘growth component’ curve. The minimum of the function is achieved very quickly as a function of iteration number; however, numerous additional iterations are required to achieve the global minimum.
**Figure S2.** Example of parameter search and convergence as a function of iteration number. Three example parameters are shown.
**Figure S3.** Application of the two models based on Eqns 3 and 6 to the irrigation experiment in Scots pine. Left panels) Application of the coupled models for control (top) and irrigated (bottom) trees. Green curve, raw bark thickness readings from the point dendrometers; black curve, ‘growth component’ curve from which tension‐driven elastic changes have been subtracted from; red curve, plastic irreversible growth. Right panels) Averages across the sampled period of the 24 h cycles of the two elastic curves, that is, tension‐driven bark water content changes (blue curve), and osmotic‐driven bark water content changes (green curve). The bands around the blue and green curves give one standard error around the mean.
**Figure S4.** Same as for Fig. S3, but applied to the stem girdling study on *Eucalyptus tereticornis*. Plots are given for the two positions (above and below the point of girdling) and two time intervals (before and after girdling)
Figure S5. Test of model performance in a field stem‐girdling experiment on *Eucalyptus globulus* at Richmond, New South Wales, Australia. This figure replicates data given in Fig. 5 of the main paper but with the data now split to show the two positions separately for the control and the mildly droughted trees. Panels on the top provide environmental information for three periods throughout the summer of 2014 (days of year 100 to 107, 126–133 and 138–145). Panels represent photosynthetically active radiation PAR, air relative humidity RH and soil relative water content SWC (control trees in blue; partially droughted and rewatered trees in red; different line types represent different depths). The first period represent the initial phase of the experiment, when three trees were mildly droughted and three were continuously watered. The second period represents the second phase of the experiment when the mildly droughted trees were re‐watered. The third period represents the post‐girdling phase, three days after the girdling had been carried out. The bottom panels give the mean inferred daily patterns of growth, for two positions along the stem (top and bottom) and separately for control (Con) and dry‐down trees (Dry). Growth for the control is given in blue (base) and violet (top); growth for the dry‐down is given in red (base) and pink (top). The ‘top’ stem position corresponds to a point above girdling; the ‘bottom’ position corresponds to a point below girdling. The figure shows that no differences exist between the mildly droughted and the control trees for either position and either period. Only the boundaries of the standard error bands for each

Supporting info item

Supporting info item
