# Supplementary material for: An empirical method that separates irreversible stem radial growth from bark water content changes in trees: theory and case studies
Source: Plant Cell Environ. 2017 Jan 6;40(2):290–303. doi: 10.1111/pce.12863 (PMC6849533; doi:10.1111/pce.12863)
Supplement: Supplementary file 1 — Supporting info item [file PCE-40-290-s001.docx]

**An empirical method that separates irreversible stem radial growth from bark water content changes in trees: theory and case studies**

**Maurizio Mencuccini, Yann Salmon, Patrick Mitchell, Teemu Hölttä, Brendan Choat, Patrick Meir, Anthony O’Grady, David Tissue, Roman Zweifel, Sanna Sevanto and Sebastian Pfautsch.**

**Supplementary Materials**

In a previous publication (Mencuccini et al., 2013), we showed that time series of bark and xylem diameter change can be used to determine bark water use. Under conditions of no radial growth (or when the time series is detrended), the equations presented in Mencuccini et al. (2013) are mathematically correct. Under conditions of radial growth however, two more processes co-occur with bark hydraulic capacitance. Without further information, elastic thickness changes driven by osmotic content changes in the bark cannot be separated from the irreversible expansion caused by deposition and growth of new xylem and phloem cells. The resulting signal is the sum of the two components, elastic and plastic (Chan et al., 2015). To separate the elastic, osmotically-driven changes from the plastic, growth-driven changes, additional empirical information and a new theoretical treatment is needed.

**Note 1. Details of the bark water use model**

Equations S1 to S5 below are consistent with those in Mencuccini et al. (2013). We assume that the entire volume change in the bark is due to change in water content and we use Hooke’s law combined with Darcy’s law for flow through porous media. However, a slight change of notation is necessary here to maintain consistency within the present paper. Here we will employ throughout the notation $\frac{dD_{xyl}^{E}}{dt}$ instead of $\frac{dD_{b}}{dt}$, to designate the predicted elastic xylem tension-driven changes in bark thickness.

Hence:

$\frac{dD_{xyl}^{E}}{dt}=L \left(\Psi_{x}-\left(P_{b}-\Pi_{b}\right)\right)$ (S1)

*L* is the hydraulic conductance of the cross-sectional contact area *A* between the xylem and phloem, (*P_b_ - Π_b_*) indicate the water potential in the bark and the reflection coefficient is assumed 1.00. It is also useful to express the water potential terms at a reference time :

$\frac{dD_{xyl}^{E*}}{dt}=L \left(\Psi_{x}^{*}-\left(P_{b}^{*}-\Pi_{b}^{*}\right)\right)$ (S2)

where all variables labelled with the * symbol are determined at a reference time. Subtracting (S2) from (S1) side by side and re-arranging, yields:

$\frac{dD_{xyl}^{E}}{dt}=L \left(\Delta\Psi_{x}-(\Delta P_{b})+\left(\Delta\Pi_{b}\right)\right)+\frac{dD_{xyl}^{E*}}{dt}$ (S3)

where $\Delta\Psi_{x}=\Psi_{x}-\Psi_{x}^{*}$, $\Delta P_{b}=P_{b}-P_{b}^{*}$ and$\Delta\Pi=\Pi_{b}-\Pi_{b}^{*}$.

Using Hooke’s law, we now express the pressure terms as a function of the measured thickness differences from the reference state (measured quantities being indicated with a caret):

$\Delta P_{b}=\frac{\varepsilon_{r,b}}{D_{b}^{*}}\Delta\hat{D}{}_{b}$ (S4)

where $D_{b}^{*}$ (m) is the inner bark diameter at a reference pressure and. As per Mencuccini et al. (2013), we assume for the moment that changes in osmotic pressure do not occur. Since the term $\Delta\Pi$ vanishes, substituting Eqns. (S4) into (S3), rearranging and simplifying, one obtains:

(S5)

Where we employ the equality $V_{b}^{*}$ =*A* $D_{b}^{*}$ and $V_{b}^{*}$ (m^3^) is the inner bark volume at a reference pressure. Osmotic potential of course can vary over time and this is dealt with in Note 2 of these Supplementary Materials. Let’s assume now that estimates of local xylem water potential $\Psi_{x}$are obtained using an Ohm’s law analogy, i.e.

(S6)

where $\hat{J}_{s}$is the locally measured axial sap flux density (or sap velocity) determined using a heat tracing technique and $\Psi_{s}$and $K_{\text{spl}}$are, respectively, the soil water potential and soil-to-plant xylem hydraulic conductance. Note that the linearity of the equation above is broadly correct, since we are only employing it to estimate the local xylem water potential at the point of sap flow measurement. Expressing the quantities at a reference time using the usual * symbol, substituting (S6) into (S5), rearranging and simplifying yields:

(S7)

The *K*_spl_ and ΔΨ_s_ are not necessarily constant but, like *L*, can vary from day to day to reflect dynamic changes in soil water potential, soil-to-plant hydraulic conductance and radial hydraulic conductance. Equation (S7) equates to:

$\frac{dD_{xyl}^{E}}{dt}=\alpha\left(\beta\text{ }J_{s}-\Delta D_{b}\right)+\gamma$ (S8)

This expression is formally identical to the one given in Mencuccini et al., (2013), except for the different notation on the left hand side of the equation, where:

α = $\frac{1}{\tau}=\frac{L A}{V_{b}^{*}}\varepsilon_{r,b}=\frac{L}{D_{b}^{*}}\varepsilon_{r,b}$*,*

β = $- \frac{D_{b}^{*}}{K_{spl} \varepsilon_{r,b}}$ and

$$\gamma=\frac{dD_{xyl}^{E*}}{dt}+L\frac{J_{s}^{*}}{K_{spl}^{*}}+L \Delta\Psi_{s}$$



And α, β and γ can vary from day to day. From the definitions of α and β above, $\text{α} \text{β}=-\frac{L}{K_{pl}}$*,* i.e., the ratio of radial to axial hydraulic conductances relative to an initial reference time.

**Note 2. Details of the osmotic model.**

***Growth model***

A diagram of model structure is given in Figure 2 of the main text. The tissues included in the live bark of trees (phloem, cambium and cambial growing region on the phloem and xylem sides) are represented by a single compartment, referred to as ‘bark’. Outside of the cambial growing region on the xylem side, tree tissues extend towards mature xylem cells. Let *n*_b_ be the volume-averaged number of moles of carbohydrates in the bark (Figure 1). The net carbohydrate flux into the bark at any height is the difference *F*_phl_ between the gross incoming flux from the canopy and the gross outflowing flux towards the roots via the transport phloem. This flux to the cambial growing regions delivers a carbohydrate flux *F*_gr_ laid down in the new cells on phloem and xylem sides. Changes in the content of solutes of the bark are entirely caused by changes in carbohydrate content.

The rate of phloem carbohydrate supply to the growing cells $F_{gr}=a n_{b}$ is assumed for simplicity to be a first-order process dependent on number of moles of carbohydrates in the bark *n*_b_, where *a* is the rate of supply of carbohydrates to the cambium per unit of number of moles of carbohydrates in the cambium (% s^-1^). This reflects the direct dependency of growth rates on carbohydrate supply and, indirectly, its potential dependence on factors such as temperature via changes in *a*. The rate of cell division/enlargement of a bark segment of volume *V*=*DA*, (i.e., equivalent to a xylem plus phloem irreversible radial diameter *D* growth over a constant vertical area *A*) is proportional to phloem carbohydrate supply to the growing cells: $\frac{dD^{P}}{dt}=k F_{gr}/A=ka n_{b}/A$, where *k/A* is the phloem plus xylem diameter growth per unit of carbohydrate supply. The dependency of cellular division/ enlargement $\frac{dD^{P}}{dt}$ on radial solute fluxes *F*_gr_ makes it possible for these fluxes to be controlled by turgor changes and therefore the strength of carbohydrate sink demand, via *k*. Parameter *k* is a lumped parameter, effectively proportional to terms in the Lockhart equation, but also incorporating respiratory losses associated with growth. The relationship embodies the two major factors controlling tree volumetric growth, i.e., carbohydrate supply (source strength) and cellular turgor in the cambial growing regions (sink strength). This approach makes no assumptions about how levels of carbohydrate supply or turgor change over time. Rather, it derives growth from the best fit to the thickness and sap flow data, allowing one to infer what the likely levels of carbohydrate availability or turgor must have been to be consistent with the observations. Changes in the number of moles of carbohydrates of the bark are then:

$\frac{dn_{b}}{dt}=F_{phl}-F_{gr}\to\frac{dn_{b}}{dt}=F_{phl}-a n_{b}$ (S9)

This first-order differential equation in *t* and $n_{b}$ can be integrated and solved for $n_{b}$ and its derivative $\frac{dn_{b}}{dt}$:

$n_{b}=n_{0}e^{-at}+\frac{F_{phl}}{a} \left( 1-e^{-at} \right)$ (S10)

$\frac{dn_{b}}{dt}=\left( F_{phl}-F_{gr,0} \right)e^{-at}$ (S11)

Where *n*_0_ and $F_{gr,0}$ are the number of moles of carbohydrates in the bark and carbohydrate flux to the cambium at time *t*=0, respectively. Elastic bark diameter changes $\frac{dD_{osm}^{E}}{dt}$ depend only on the changes in the number of moles of carbohydrates $\frac{dn_{b}}{dt}$ following van’t Hoff relationship Ψ_Π_=-*n*_b_*R T*/*V,* where Ψ_Π_, *T* and *R* are the osmotic potential, the absolute temperature and the gas constant, respectively. It is assumed that bark volume *V* represents only the symplastic volume. Because changes caused by xylem tension are accounted for separately (cf., Note 1), osmotically-driven changes are caused only by changes in the number of moles of carbohydrates, hence:

$\frac{d\Psi_{\Pi}}{dt}=\frac{\delta\Psi_{\Pi}}{\delta n_{b}} \frac{dn_{b}}{dt} = \frac{-R T}{V_{b}^{*}} \frac{dn_{b}}{dt}$ (S12)

Equally, at constant total water potential, $\frac{d\Psi_{x}}{dt} =0$:

$\frac{dP_{b}}{dt} = -\frac{d\Psi_{\Pi}}{dt}$ (S13)
and
 $\frac{dP_{b}}{dt} = \frac{\varepsilon_{r,b}}{D_{osm}^{E*}}\frac{dD_{osm}^{E}}{dt}$ (S14)

from Hooke’s law. Combining (S12), (S13) and (S14) leads to:

$\frac{dn_{b}}{dt} =\frac{\varepsilon_{r,b} A}{R T}\frac{dD_{osm}^{E}}{dt}$ (S15)
Therefore, the final expression for the osmotically-driven changes in elastic bark volume over time is:

$\frac{dD_{osm}^{E}}{dt} =\frac{R T}{\varepsilon_{r,b} A}\frac{dn_{b}}{dt}$ (S16)

Using (S10) and (S11), the changes in elastic $D_{osm}^{E}$ and the irreversible *D*^P^ thickness can now be linked to time and fluxes as:

$\frac{dD_{osm}^{E}}{dt}=\frac{R T}{\varepsilon_{r,b} A}\frac{dn_{b}}{dt}=\frac{R T}{\varepsilon_{r,b} A}\left( F_{phl}-F_{gr,0} \right)e^{-at}$ (S17)

$\frac{dD^{P}}{dt}= {\frac{k}{A}F}_{gr}=\frac{k}{A} a n_{b}=\frac{k}{A} \left( F_{gr,0} e^{-at}+F_{phl} \left( 1-e^{-at} \right) \right)$ (S18)

Eqns. (S17) and (S18) can be solved simultaneously to eliminate the unknown phloem supply rate $F_{phl}$:

$\frac{dD^{P}}{dt}=\frac{k}{A}F_{gr,0}+ \frac{\varepsilon_{r,b} k}{RT}(e^{at}-1)\frac{dD_{osm}^{E}}{dt}\to\frac{dD^{P}}{dt}=m+r\frac{dD_{osm}^{E}}{dt}$ (S19)

where $m= \frac{k}{A}F_{\text{gr,0}}$ is the combined rate of phloem and xylem diameter growth at the beginning of each measurement period. Assuming a certain periodicity in the dendrometer measurements (e.g., every 5 minutes), then, *t*=5min=*t**, and $r=\frac{\varepsilon_{r,b} k}{RT}(e^{at^{*}}-1)=\frac{\varepsilon_{r,b} k}{RT}l$, where *l* is a scalar (unitless) constant for each setup. Parameter *r* depends primarily on *a*, the product between $\varepsilon_{r,b}$ the bark elastic modulus and *k* the rate of growth per unit of carbohydrate flux (i.e., the two parameters controlling elastic and irreversible changes) over the time interval of the measurements. It reflects the sensitivity of irreversible volume changes to elastic volume changes. In terms of their dependency on environmental variables, *m* should be sensitive primarily to turgor (via *k*) and *r* to both turgor and temperature (via *k* and *a*, respectively). Therefore, a linear relationship exists between elastic $\frac{dD_{osm}^{E}}{dt}$ and plastic $\frac{dD^{P}}{dt}$ changes in bark diameter driven by the initial changes imposed upon by $F_{gr,0}$. Therefore, as wanted in Eqn. (5) of the main text, expressions can be obtained for the changes in plastic and elastic diameter as a function only of measured changes in total bark diameters (corrected for bark water content changes). These expressions are conditional on three assumptions, i.e., that a) the carbohydrate flux to the growing cells depends as a first-order process on bark carbohydrate content, b) that the rate of cell division/enlargement in the bark is proportional to phloem carbohydrate flux to the cambium for each time interval, and c) that the bark is treated as symplastic volume only.

An equivalent expression is obtained if one solves the system by eliminating $F_{gr}$ while retaining $F_{phl}$, i.e.:

$\frac{dD^{P}}{dt}=F_{ph} \left( 2e^{-at}-1 \right)-\frac{\varepsilon_{r,b} k}{RT}\frac{dD_{osm}^{E}}{dt}$ (S20)

In practice, to avoid making strong assumptions on the constancy of any of the 'parameters' in the model (as conditions may vary from time to time), it is assumed that they are constant only for relatively short periods of time during the day. They are instead allowed to vary across times of day and across days (but, in this case, following the same within-day pattern times a multiplier). Within each interval (and given enough days of measurements), there are then enough replicated points that one can estimate the values of the parameters within a certain error. As an example, *m* in Eqn. (S19) is assumed constant for, say, 6 hours and allowed to vary for the next interval of 6 hours. The within-day pattern (i.e., the changes in *m* from interval to interval across the 24-hour cycle) is assumed constant across days (times a daily multiplier –which can also be zero). This is done because the primary interest here is in estimating the mean within-day pattern. Therefore, the multiple days of measurements are considered replicates (within some error) of the same mean daily pattern.
