# Supplementary material for: An empirical method that separates irreversible stem radial growth from bark water content changes in trees: theory and case studies
Source: Plant Cell Environ. 2017 Jan 6;40(2):290–303. doi: 10.1111/pce.12863 (PMC6849533; doi:10.1111/pce.12863)
Supplement: Supplementary file 2 — Supporting info item [file PCE-40-290-s002.pptx]

## Slide 1
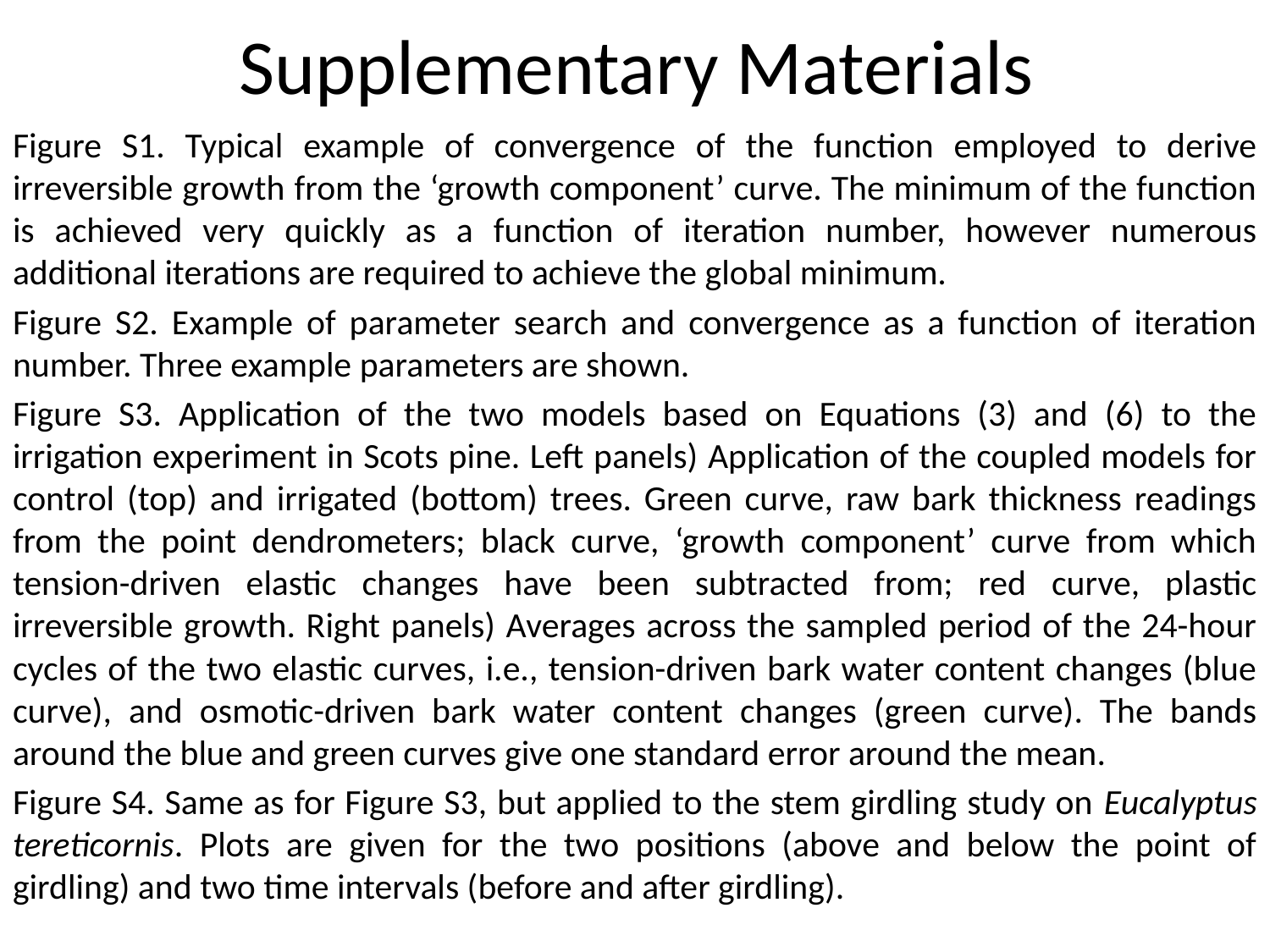

# Supplementary Materials
Figure S1. Typical example of convergence of the function employed to derive irreversible growth from the ‘growth component’ curve. The minimum of the function is achieved very quickly as a function of iteration number, however numerous additional iterations are required to achieve the global minimum.
Figure S2. Example of parameter search and convergence as a function of iteration number. Three example parameters are shown.
Figure S3. Application of the two models based on Equations (3) and (6) to the irrigation experiment in Scots pine. Left panels) Application of the coupled models for control (top) and irrigated (bottom) trees. Green curve, raw bark thickness readings from the point dendrometers; black curve, ‘growth component’ curve from which tension-driven elastic changes have been subtracted from; red curve, plastic irreversible growth. Right panels) Averages across the sampled period of the 24-hour cycles of the two elastic curves, i.e., tension-driven bark water content changes (blue curve), and osmotic-driven bark water content changes (green curve). The bands around the blue and green curves give one standard error around the mean.
Figure S4. Same as for Figure S3, but applied to the stem girdling study on Eucalyptus tereticornis. Plots are given for the two positions (above and below the point of girdling) and two time intervals (before and after girdling).

## Slide 2
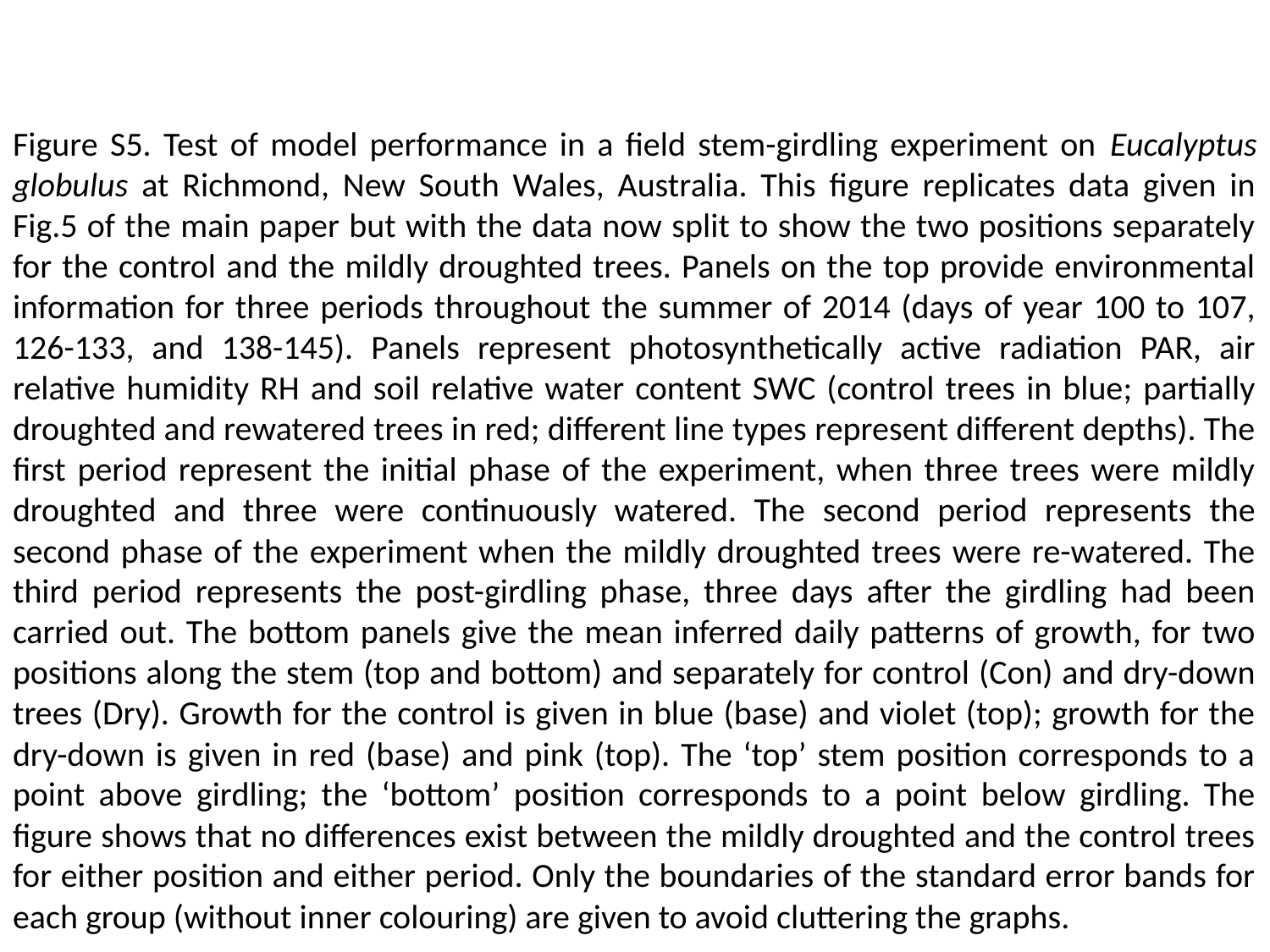

Figure S5. Test of model performance in a field stem-girdling experiment on Eucalyptus globulus at Richmond, New South Wales, Australia. This figure replicates data given in Fig.5 of the main paper but with the data now split to show the two positions separately for the control and the mildly droughted trees. Panels on the top provide environmental information for three periods throughout the summer of 2014 (days of year 100 to 107, 126-133, and 138-145). Panels represent photosynthetically active radiation PAR, air relative humidity RH and soil relative water content SWC (control trees in blue; partially droughted and rewatered trees in red; different line types represent different depths). The first period represent the initial phase of the experiment, when three trees were mildly droughted and three were continuously watered. The second period represents the second phase of the experiment when the mildly droughted trees were re-watered. The third period represents the post-girdling phase, three days after the girdling had been carried out. The bottom panels give the mean inferred daily patterns of growth, for two positions along the stem (top and bottom) and separately for control (Con) and dry-down trees (Dry). Growth for the control is given in blue (base) and violet (top); growth for the dry-down is given in red (base) and pink (top). The ‘top’ stem position corresponds to a point above girdling; the ‘bottom’ position corresponds to a point below girdling. The figure shows that no differences exist between the mildly droughted and the control trees for either position and either period. Only the boundaries of the standard error bands for each group (without inner colouring) are given to avoid cluttering the graphs.

## Slide 3
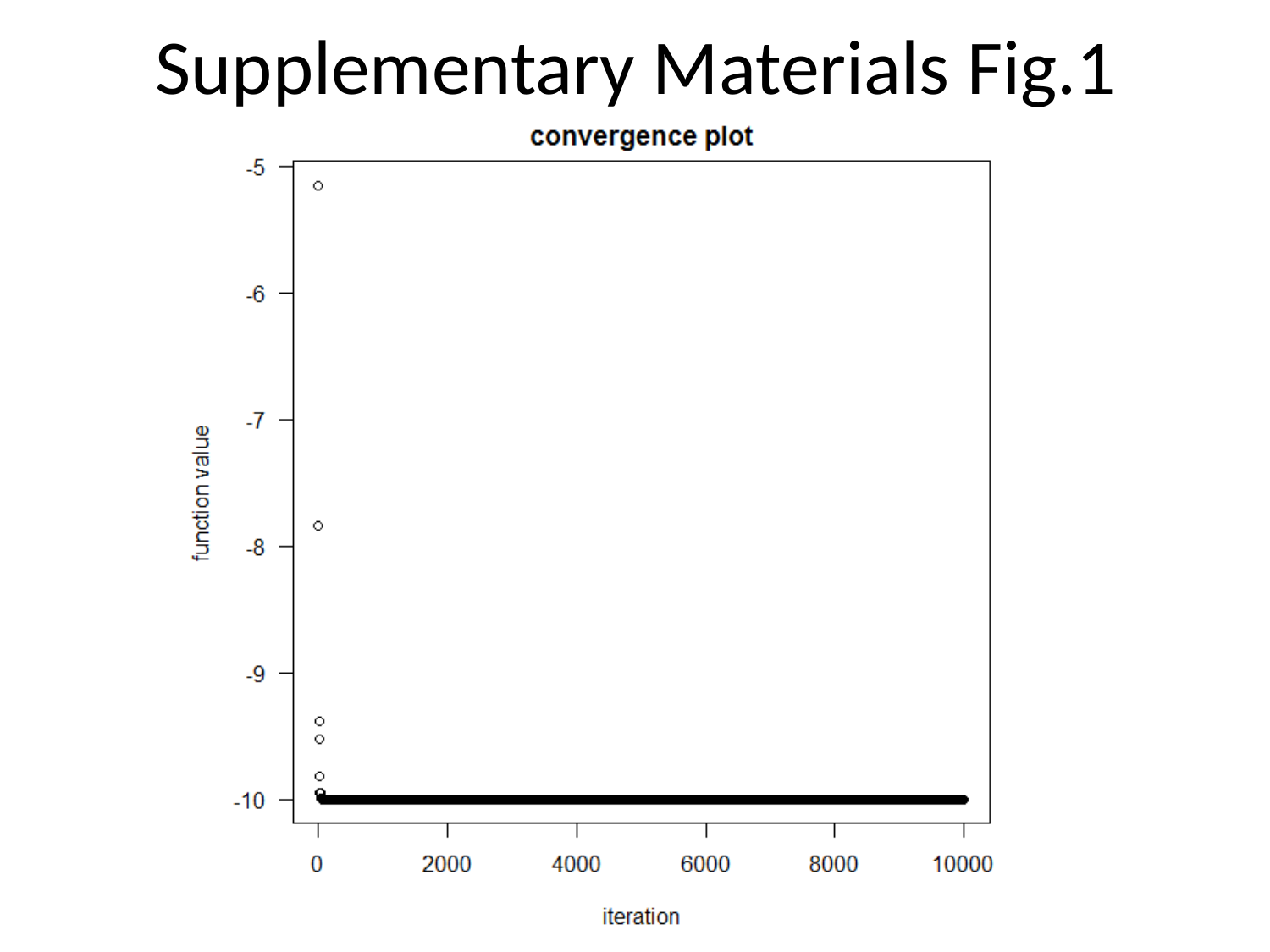

# Supplementary Materials Fig.1

## Slide 4
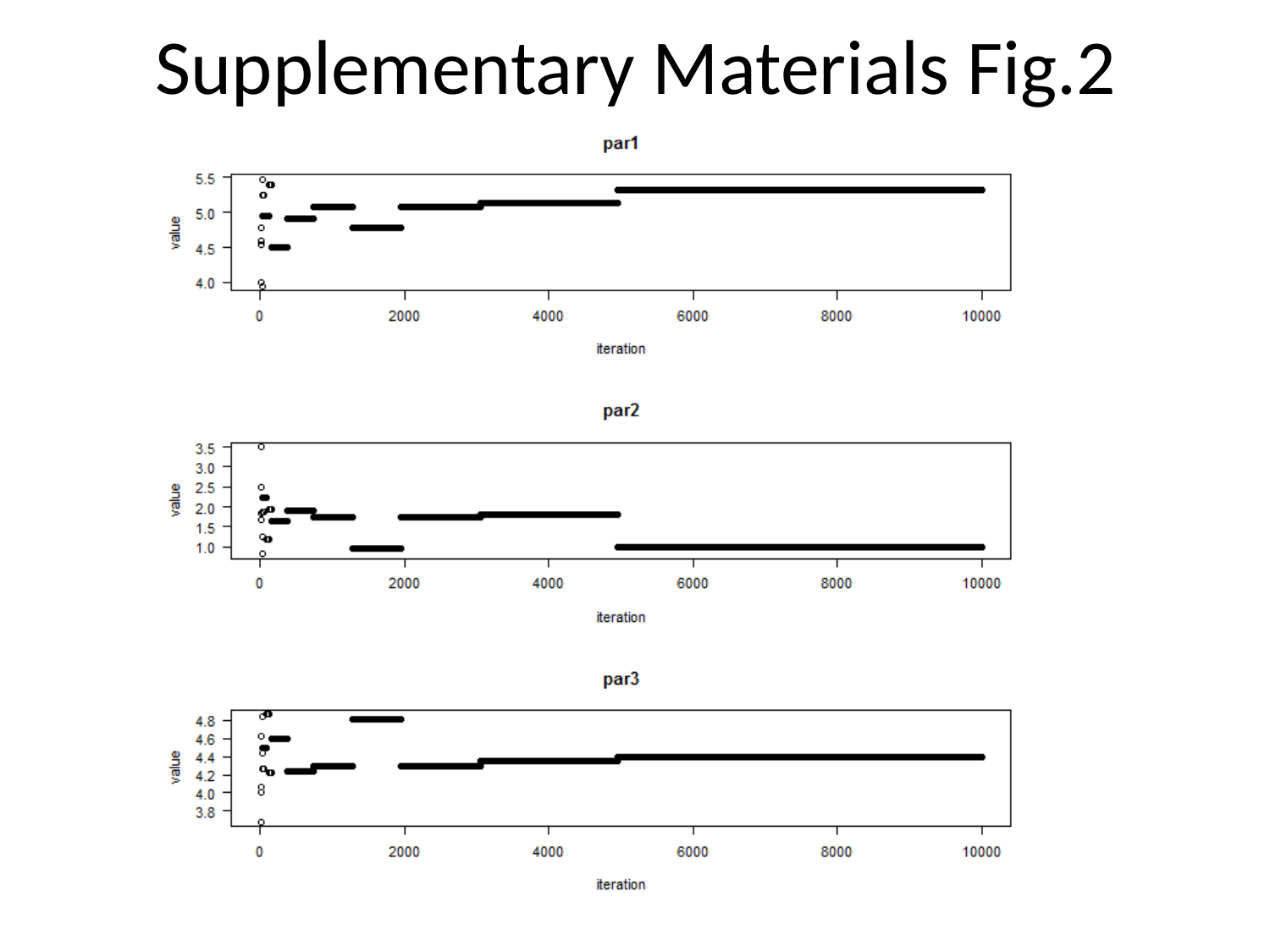

# Supplementary Materials Fig.2

## Slide 5
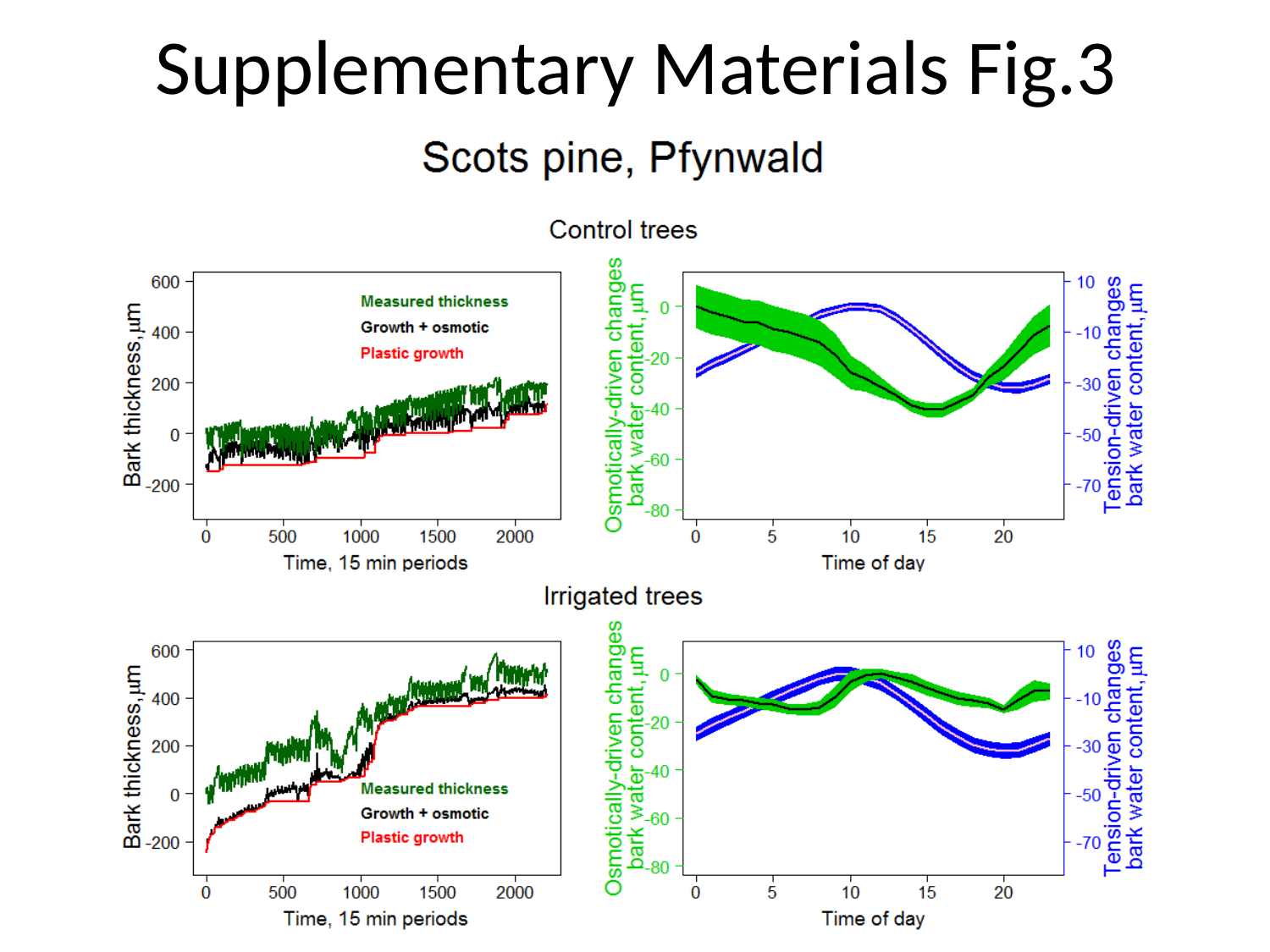

# Supplementary Materials Fig.3

## Slide 6
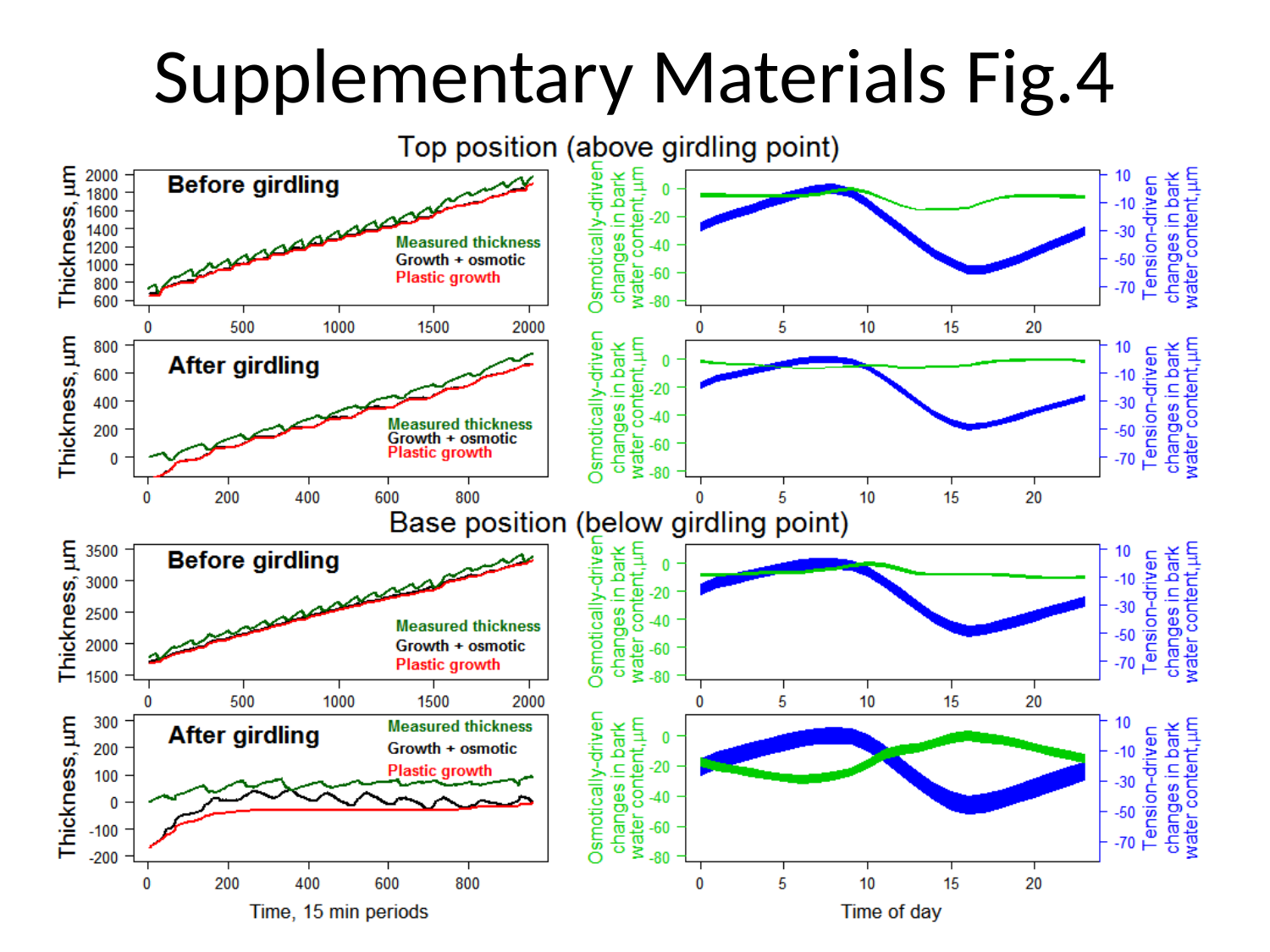

# Supplementary Materials Fig.4

## Slide 7
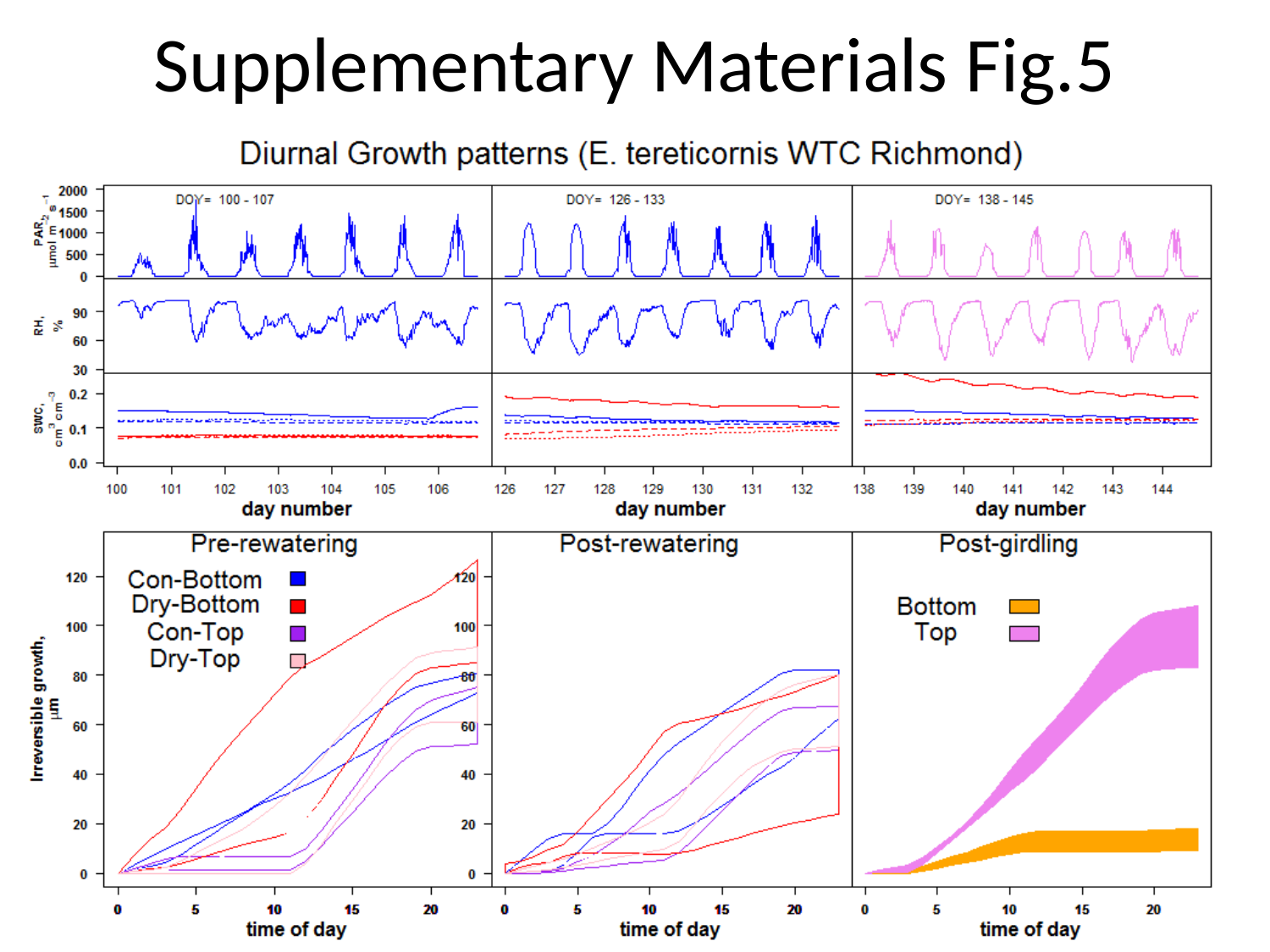

# Supplementary Materials Fig.5
